# Shoot and root insect herbivory change the plant rhizosphere microbiome and affects cabbage–insect interactions through plant–soil feedback

**DOI:** 10.1111/nph.17746

**Published:** 2021-10-09

**Authors:** Julia Friman, Peter N. Karssemeijer, Julian Haller, Kris de Kreek, Joop J.A. van Loon, Marcel Dicke

**Affiliations:** ^1^ Laboratory of Entomology Wageningen University and Research Droevendaalsesteeg 1 Wageningen 6708 PB the Netherlands

**Keywords:** Brussels sprouts (*Brassica oleracea*), Cabbage root fly (*Delia radicum*), plant defense, plant–soil feedback, plant‐mediated interactions, rhizosphere microbiome

## Abstract

- Plant–soil feedback (PSF) may influence plant–insect interactions. Although plant defense differs between shoot and root tissues, few studies have examined root‐feeding insect herbivores in a PSF context. We examined here how plant growth and resistance against root‐feeding *Delia radicum* larvae was influenced by PSF.
- We conditioned soil with cabbage plants that were infested with herbivores that affect *D*. *radicum* through plant‐mediated effects: leaf‐feeding *Plutella xylostella* caterpillars and *Brevicoryne brassicae* aphids, root‐feeding *D. radicum* larvae, and/or added rhizobacterium *Pseudomonas simiae* WCS417r. We analyzed the rhizosphere microbial community, and in a second set of conspecific plants exposed to conditioned soil, we assessed growth, expression of defense‐related genes, and *D*. *radicum* performance.
- The rhizosphere microbiome differed mainly between shoot and root herbivory treatments. Addition of *Pseudomonas simiae* did not influence rhizosphere microbiome composition. Plant shoot biomass, gene expression, and plant resistance against *D*. *radicum* larvae was affected by PSF in a treatment‐specific manner. Soil conditioning overall reduced plant shoot biomass, *Pseudomonas simiae*‐amended soil causing the largest growth reduction.
- In conclusion, shoot and root insect herbivores alter the rhizosphere microbiome differently, with consequences for growth and resistance of plants subsequently exposed to conditioned soil.

## Introduction

Plants are members of complex communities, in which they interact with a plethora of other organisms such as insects and microbes (van der Heijden *et al*., 2008; Berendsen *et al*., 2012; Stam *et al*., 2014). Plant responses to the biotic or abiotic environment can affect many of these interactions and can shape the roots and their associated microbiome (Sasse *et al*., 2018; Stringlis *et al*., 2019; Wang *et al*., 2019; Delory *et al*., 2020; Kostenko & Bezemer, 2020). Shaping of the root‐associated microbial community may impact future plants growing in the same soil. The net effect of all biotic and abiotic properties of soil conditioned by plants that previously grew in it on plants subsequently growing in the same soil is called plant–soil feedback (PSF) (van der Putten *et al*., 2013; Kaplan *et al*., 2018; Bennett & Klironomos, 2019). PSF can affect the performance of plants positively (Kulmatiski *et al*., 2017) or negatively (Ma *et al*., 2017; Lekberg *et al*., 2018). Although an increasing number of studies focuses on the effects of PSF on plant growth, its effect on plant resistance is less explored, in particular plant defense against belowground insect herbivores (Hu *et al*., 2018).

Plants possess interconnected hormonal signaling pathways that respond to insect herbivory in both shoot and root tissue. Plant defenses to insect herbivores are mainly regulated by the phytohormones jasmonic acid (JA) and salicylic acid (SA), but also other plant hormones such as abscisic acid (ABA) and ethylene (ET) are involved (Erb *et al*., 2012b; Verma *et al*., 2016). Plants respond to herbivory by upregulating primarily JA‐ or SA‐associated signaling depending on the attacking insect species. Chewing insects generally induce JA production, whereas phloem‐feeding insects induce SA biosynthesis (Erb *et al*., 2012b; Stam *et al*., 2014).

There are differences in plant defense and phytohormone regulation between plant shoot and root tissues (Johnson *et al*., 2016). For instance, levels of the defensive glucosinolates in brassicaceous plants differ substantially between shoots and roots (Tsunoda *et al*., 2017). In terms of phytohormonal signaling, JA is thought to be less inducible in roots compared to shoots (Erb *et al*., 2012a; Tytgat *et al*., 2013), but increased levels do occur after herbivore attack (Erb *et al*., 2009; Lu *et al*., 2015; Karssemeijer *et al*., 2020), and SA may serve different functions in root and shoot tissues (Erb *et al*., 2012a; Lu *et al*., 2015).

Plant hormones do not only govern plant defense, they also influence root exudates and therefore consequently the microbiome around the plant root (Carvalhais *et al*., 2015; Eichmann *et al*., 2021). Therefore it is not surprising that feeding by shoot and root herbivores induces microbiome alterations, through altered plant root exudation (Dawson *et al*., 2004; Kostenko *et al*., 2012; Kim *et al*., 2016; Kong *et al*., 2016; Ourry *et al*., 2018; Friman *et al*., 2021b). Herbivores can also influence the soil microbiome directly, for instance through caterpillar frass or aphid honeydew that mixes with soil (Frost & Hunter, 2004). The resulting changes in microbiome and soil properties can affect the chemical composition of subsequently growing plants (Meiners *et al*., 2017) which in turn can affect herbivorous insects (Kostenko *et al*., 2012). In this manner, phytohormone‐mediated signaling pathways and by extension plant defense relying on types and levels of secondary metabolites, can be modified by PSF (Ma *et al*., 2017; Hu *et al*., 2018; Zhu *et al*., 2018; Bennett & Klironomos, 2019). For instance, caterpillars of the cabbage moth *Mamestra brassicae* showed decreased performance when feeding on plants grown in soil conditioned by plants infested by root‐feeding wireworms *Agriotes lineatus*, compared to caterpillars feeding on plants grown in soil conditioned by caterpillar‐infested plants (Kostenko *et al*., 2012). Thus, herbivores can affect plant defense through PSF, and the identity of the herbivore species in the conditioning phase may be an important factor. Because plants respond differently to insect herbivores depending on their feeding guild and feeding site, it is plausible that different types of insects cause different changes to the plant‐associated microbe community. Whether the underlying microbial community changes are comparable between insect feeding guilds and feeding location has received little attention so far.

Some root‐associated bacteria are known to boost plant growth, and consequently have been coined plant‐growth‐promoting rhizobacteria (PGPR). A number of these PGPR can induce systemic resistance (ISR) in the plant, a mechanism that enhances resistance against a range of plant attackers (Pineda *et al*., 2010; Pieterse *et al*., 2014; Friman *et al*., 2021b). These ISR‐inducing bacteria can mediate PSF. *Arabidopsis thaliana* recruited an assemblage of ISR‐inducing microorganisms after infection with downy mildew, *Hyaloperonospora arabidopsidis*, which subsequently increased plant resistance of plants grown in the same soil against the same pathogen (Berendsen *et al*., 2018). Although plant‐growth‐promoting microbes are known to modulate plant resistance against insects (Pineda *et al*., 2010), it remains to be investigated how these rhizobacteria affect plant defense against insects in plant conspecifics growing in the same soil.

Here, we studied how shoot‐ and root‐feeding insect herbivores and beneficial rhizobacteria affect the rhizosphere microbiome, and how these differences through PSF affect plant growth and defense against a root herbivore in plants subsequently growing in the same soil. We conditioned soil by growing *Brassica oleracea* plants induced by either root‐chewing *Delia radicum*, leaf‐chewing *Plutella xylostella,* phloem‐feeding *Brevicoryne brassicae*, or by adding growth‐promoting and ISR‐inducing PGPR *Pseudomonas simiae* WCS417r to the soil. These inducers have previously been tested for their influence on *D. radicum* performance through plant‐mediated effects, where *Plutella* *xylostella* negatively influenced *D. radicum* performance, *Brevicoryne* *brassicae* had no effect (Karssemeijer *et al*., 2020), and *Pseudomonas* *simiae* positively affected the insect (Friman *et al*., 2021a). After removal of the conditioning plants and insects, we used a mixture of sterilized and conditioned soil to grow a consecutive set of *Brassica* *oleracea* plants, for which we assessed growth, defense‐related gene expression, and resistance against the root herbivore *D. radicum*. We aimed to elucidate the effect of the inducers on the rhizosphere microbial community, and how these changes may moderate plant‐mediated interactions between biotic inducers. We hypothesized that the induction by leaf‐chewing, root‐chewing, and phloem‐feeding insect herbivores would have distinct effects on the rhizosphere microbiome due to their respective induction of different phytohormones, and that plants grown in these soils would differ in resistance against *D. radicum*. We expected that *Pseudomonas* *simiae* would increase plant growth in the feedback phase, and increase *D. radicum* performance.

## Material and Methods

### Plant growth conditions

**Fig. 1:**
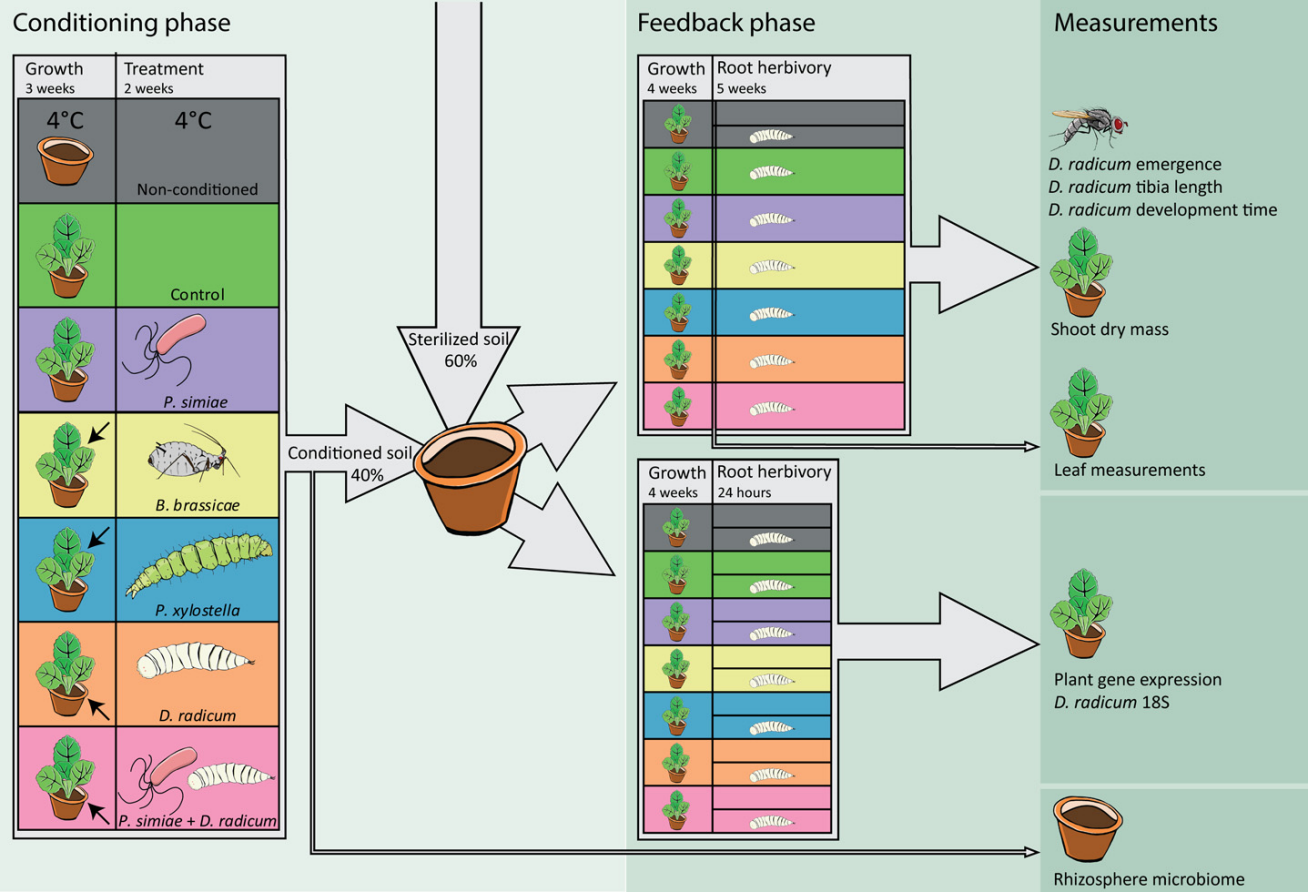
Overview of the experimental design. Soil was conditioned by *Brassica oleracea* plants that, after 3 wk of growth, were induced by six treatments represented by colored boxes in the conditioning phase. The treatments were uninfested plants (no herbivores, green), *Brevicoryne brassicae* (yellow), *Plutella xylostella* (blue), *Delia radicum* (orange), *Pseudomonas simiae* WCS417r (purple), *D. radicum* and *Pseudomonas simiae* WCS417r (pink). Arrows in the leftmost panel indicate herbivore feeding locations (shoot or root). Additionally, soil was stored at 4°C to be used as nonconditioned treatment in the feedback phase (gray). After 2 wk of induction, plants and insects were removed and rhizosphere microbiome samples were taken. The remaining soil of each treatment was mixed with sterilized soil (40 : 60, v/v). These soil mixes were used to grow two new sets of *Brassica oleracea* plants, one set was used for gene expression assessment (24 h post‐infestation) and the other set for plant and insect assessment (5 wk post‐infestation). In the feedback phase, plants were exposed to *D. radicum* root herbivory, and the performance of the root herbivore was assessed, as well as plant performance and plant defense‐related gene expression.

Our study system consisted of *Brassica oleracea*, a globally important cultivated crop plant. *Brassica oleracea* var. *gemmifera* cv. “Cyrus” seeds (Syngenta Seeds, Enkhuizen, The Netherlands) were germinated in a seeding tray with seedling soil in a glasshouse with 21 ± 3°C and 16 ± 3°C day and night temperatures respectively. Natural daylight was supplemented with 400 W metal halide lamps (200 µmol m^−2^ s^−1^ photosynthetically active radiation) when photosynthetic active radiation (PAR) dropped below 400 μmol m^−2^ s^−1^, in a 16 h : 8 h, light : dark cycle. After 3 d, plants were transplanted to 1 l pots containing potting soil and grown in glasshouse conditions for 3 wk with identical settings as earlier at 60 ± 10% relative humidity (RH). Plants were watered three times per week from the bottom until the soil was moist. Plants were additionally fertilized twice per week with 50 ml of Hyponex solution (nitrogen, phosphorus and potassium (NPK) = 7 : 6 : 19, electrical conductivity = 1.6). As the staring soil can be important in PSF experiments (French *et al*., 2021), we used the same batch of soil throughout the experiment. Seedling and potting soil from the conditioning phase was bagged and stored at 4°C for use in the feedback phase (Fig. 1).

### Insect rearing

Worldwide, the most important belowground feeding insect on *Brassica oleracea* is the specialist chewer cabbage root fly *D. radicum* L. (Diptera: Anthomyiidae). The female flies deposit a cluster of eggs in the soil near the plant stem base. After hatching, the larvae feed in the primary root. The larvae leave the root to pupate in the soil and emerge later as adult flies. Experimental *D*. *radicum* larvae were reared on rutabaga roots (*Brassica* *napus* var. *napobrassica*) at 22 ± 1°C, 70% RH and a 16 h : 8 h, light : dark cycle. The flies were caught in Zeewolde in the Netherlands in 2013 and reared in the laboratory since. Adult flies were fed honey and a 1 : 1 : 1 mix of milk powder, sugar and yeast flakes. *Plutella xylostella* L. (Lepidoptera: Plutellidae) were reared on *Brassica* *oleracea* var. *gemmifera*. Second instar larvae were used in this experiment. *Brevicoryne brassicae* L. (Hemiptera: Aphididae) were reared on *Brassica* *oleracea* var. *gemmifera*, and wingless adults were used as inducers in the experiment. These insects were reared at 22 ± 2°C, 70% RH and a 16 h : 8 h, light : dark cycle.

### *Pseudomonas simiae* WCS417r growing conditions and solution preparation

The *Pseudomonas simiae* WCS417r (formerly *Pseudomonas* *fluorescens* (Berendsen *et al*., 2015)) bacterial inoculum was prepared by incubating bacteria on King’s B (KB) medium agar plates supplemented with rifampicin (25 µg ml^−1^) for 48 h at 28°C. Cells were collected and suspended in sterilized 10 mM magnesium sulfate (MgSO_4_) solution. The suspension's optical density was adjusted to 1 × 10^9^ colony‐forming unit (CFU) ml^−1^ (OD660 = 1.0).

### Conditioning phase: induction with insects and rhizobacteria

After 3 wk of growth, plants were infested with insects and/or exposed to *Pseudomonas* *simiae* inoculum. Each treatment had 24 replicates divided over four trays with six plants placed in individual pots on saucers, to prevent sharing water between plants. Treatments were *D*. *radicum*, *D*. *radicum* plus *Pseudomonas* *simiae* WCS417r, *Plutella* *xylostella*, *Brevicoryne* *brassicae*, *Pseudomonas* *simiae* WCS417r alone and control plants (Fig. 1). Control plants were noninfested and noninoculated. For infestation with *Plutella* *xylostella* (L2) or *Brevicoryne* *brassicae* (apterous adults), 10 individuals were carefully transferred to the fourth leaf counted along the stem from the stem base to their respective treatment. To prevent insect contamination between the treatments, the petiole of the infested leaf was wrapped in cotton wool, bagged in a net and fixed with a piece of metal wire. The fourth leaves of the control plants were also wrapped in a similar manner. *Delia radicum* neonates were brushed on the carefully exposed stem base, just below soil level. For treatments that received *Pseudomonas* *simiae* WCS417r, bacterial suspension was applied next to the stem with a syringe. Each pot received 20 ml solution, which equals 2 × 10^10^ CFU, and 8 × 10^7^ CFU g^−1^ of soil. Control plants received 20 ml of sterilized 10 mM MgSO_4_, applied in a similar manner as treatment plants.

### Conditioning phase: soil and microbiome collection

Plants were exposed to insects and rhizobacterial inoculation for 2 wk. Aboveground plant parts and primary roots were then removed from the soil. For soil microbiome analysis, *c*. 3 g of secondary roots and root‐attached soil were pooled from the six plants in each tray. Thus, the six plants in each tray were considered one biological replicate. Pooled roots were collected in 50 ml tubes containing 25 ml of sterilized buffer solution (6.33 g l^−1^ NaH_2_PO_4_ and 10.96 g l^−1^ NaH_2_PO_4_ × 2H_2_O). Tubes were vigorously shaken for 30 s, and centrifuged for 7 min at 3700 ***g***. Supernatant was removed, as well as large chunks of root with sterilized tweezers. The soil slurry was transferred with a sterilized spoon into 1.5 ml tubes, and centrifuged for 5 min at 11 000 ***g***. Supernatant was removed and samples were then stored at −80°C. After taking microbiome samples, soils of all plants from the same treatment were homogenized by mixing by hand, using clean gloves for each treatment. For soils conditioned with plants infested with *D*. *radicum*, special care was taken to remove any larvae from the soil.

### Feedback phase: setup and measurements

Soil from the conditioning phase was mixed with γ‐irradiated soil (> 25 kGy; Steris, Ede, the Netherlands) in a ratio of 40% conditioned soil: 60% sterilized soil (v/v). The soil mixture was divided over 1 l pots, into 30 replicates per feedback treatment. We are aware of the discussion between mixed soil sampling strategy and independent soil sampling strategy in PSF experiments (Reinhart & Rinella, 2016; Cahill *et al*., 2017; Gundale *et al*., 2019). Since our experiment was performed in pots with similar starting soil, we believe the discussion is less applicable to our study.

A soil treatment was added consisting of pots containing a 40 : 60 mix of sterilized soil together with the original potting soil that was used in the conditioning phase (stored for 6 wk at 4°C), to include a treatment consisting of soil with a microbiome similar to that of the soil used as starting material in the conditioning phase; this treatment is hereafter referred to as ‘nonconditioned’. *Brassica oleracea* seeds were sown on seedling soil, that had been stored at 4°C from the start of the experiment, to expose the seeds to a similar microbiome as the first set of plants. After 3 d, the seedlings were transplanted to the feedback phase pots. Plants were grown for 25 d under the same glasshouse settings as during the conditioning phase. After 1 wk of plant growth the pots were provided with sticks to later support insect nets. Plants were divided into two sets, one for gene expression analysis after 24 h of exposure to *D. radicum* larvae and the other for assessing plant and *D*. *radicum* performance.

### Feedback phase: plant and root herbivore performance

After 4 wk of growth, plants were infested with 10 neonate *D*. *radicum* larvae. Half of the plants grown on nonconditioned soil were infested with larvae, to assess effects of *D*. *radicum* on plant performance. The larval infestation was performed as described earlier. For insect performance measurements, all plants were individually covered with a mesh bag 10 d after infestation. Plants were inspected daily for emerged *D*. *radicum* adults, which were then collected, frozen, and stored at −20°C. *Delia radicum* size was determined by measuring hind tibia length with a digital microscope (Dino‐Lite Edge digital microscope, New Taipei City, Taiwan) as a proxy for fly body size (Soler *et al*., 2007; Karssemeijer *et al*., 2020). Developmental time was recorded as the time between larval infestation and adult emergence.

Plant performance in the feedback phase was assessed as leaf area of the second leaf after 3 wk of plant growth as a proxy for plant size. Since measuring the leaf area might damage the leaf, we measured only leaf width and length in experimental plants. We then calculated the leaf area from the leaf measurements using the following formula: length × width × leaf area coefficient = leaf area. The coefficient was calculated by measuring width, length, and leaf area of 10 *Brassica oleracea* nonexperimental plants’ leaves of similar size using LeafByte (Getman‐Pickering *et al*., 2020). Five weeks after infestation, the plant shoot was harvested and its biomass determined. Dry shoot biomass was recorded to the nearest 0.01 g (DK‐6200‐C‐M; Allscales, Houston, TX, USA) after drying at 105°C for 24 h.

### Plant defense‐related gene expression analysis

After 4 wk of growth on conditioned soil, half of the plants were infested with 10 neonate *D*. *radicum* larvae (Fig. 1), to assess plant defense gene expression under PSF conditions. After 24 h of infestation, primary roots were harvested by uprooting the plants, cutting off secondary roots, and freezing the primary root directly in liquid nitrogen. One leaf disk from three leaves per plant was collected with a 1 cm diameter metal puncher. Samples were pooled for three plants, and immediately frozen in liquid nitrogen to form one replicate. Samples were stored at −80°C.

Frozen samples were ground in liquid nitrogen, with a mortar and pestle for roots, or with a small pestle directly in the collection tube for leaves. Plant RNA was extracted with Isolate II Plant RNA kit (Bioline, London, UK) following the manufacturer’s instructions, and converted to complementary DNA (cDNA) (SensiFAST, Bioline). Quantitative polymerase chain reaction (qPCR) analysis was performed to test transcript levels of genes of interest (CFX96™ Real‐Time System; Bio‐Rad, Hercules, CA, USA). The primer efficiency was calculated with qPCR by determining a standard curve with a dilution series. Reference genes *SAR1a*, *Btub*, *Act‐2*, *PER4*, *GADHP* and *EF1a* were tested on 10 randomly selected samples from both roots and leaves to determine the optimal combination of reference genes using GeNorm (Vandesompele *et al*., 2002) in qbase+ v.3.1 (Biogazelle, Zwijnaarde, Belgium). For roots, *Act‐2* and *SAR1a* were used as reference genes, while for leaves *Btub* and *SAR1a* were used. We analyzed transcript levels in roots for *LOX6*, *MYB28*, *CYP81F1*, *MYB72* and *PDR9*, and in leaves for *LOX2* and *MYB28* (Supporting Information Table S1). For *MYB72* and *PDR9*, two genes studied in *Arabidopsis* (At1g56160 and At3g53480, respectively), orthologous genes in *Brassica* *oleracea* were identified using the integrative orthology finder in PLAZA (van Bel *et al*., 2018).

### *Delia radicum* biomass assessment

One of the main challenges when working with *D. radicum* is the difficulty of assessing larval performance. The larvae are small and colorless, and during the first days of feeding they dig into the root, making it difficult to get them back. To overcome this obstacle, we developed species‐specific primers (see Methods S1; Table S2; Fig. S1). These primers specifically target the 18S region of *D. radicum*, without amplifying nontargets such as those found in fungus gnats and nematodes which may occur in the experimental soil. We used these primers in the root samples collected for gene expression analysis (Fig. 1) as a proxy of larval performance and normalized the quantity relative to the plant reference genes *Act‐2* and *SAR1a*.

### Soil microbiome analysis

Total genomic DNA (gDNA) from 0.25 ± 0.01 g of pooled rhizosphere soil was extracted using the DNeasy PowerSoil Kit (Qiagen, Hilden, Germany) according to the manufacturer's instructions. The nucleic acid concentration and purity of samples were quantified with a spectrophotometer (DeNovix, Wilmington, DE, USA). For bacteria, the V4 region of the *16S* gene was amplified using the 515F/806R primers (Caporaso *et al*., 2011) (Roche FastStart High Fi, 58°C, 26 cycles). For fungi, the ITS2 region was amplified using the fITS9/ITS4R primers (Ihrmark *et al*., 2012) (Qiagen HotStarTaq, 52°C, 33 cycles). Microbial DNA was sequenced by Illumina MiSeq, 250 bp paired‐end, to a depth of 79 138 to 166 482 reads per sample. Amplification, library preparation and sequencing were performed by Génome Québec (Montreal, QC, Canada). Raw sequencing data are available from the European Nucleotide Archive (https://www.ebi.ac.uk/ena/), under study accession number PRJEB47452.

Raw fastq files were processed using Cutadapt (Martin, 2011) and the Dada2 pipeline (Callahan *et al*., 2016). The code used for sample processing is available in the Notes S1. After processing, 62 735 to 97 854 bacterial reads and 47 339 to 98 457 fungal reads remained per sample. Taxonomy was assigned using the silva v.138 database (Quast *et al*., 2013) for bacteria and the unite v.8.2 database (Nilsson *et al*., 2018) for fungi. We filtered ASVs (amplicon sequence variants) with too few occurrences using the effective sample approach in metagenomeSeq (Paulson *et al*., 2013).

### Statistical analysis

Statistical analysis was performed in R, v.4.0.0 (R Core Team, 2018), with Rstudio v.1.2.5042. For microbiome analysis, counts were normalized using metagenomeSeq (Paulson *et al*., 2013). Principle coordinate analysis (PCoA) was performed using Bray–Curtis dissimilarity in phyloseq (McMurdie & Holmes, 2013). Permutational multivariate analysis of variance (PERMANOVA) was done with 99 999 permutations using Bray–Curtis dissimilarity with the adonis function (Oksanen *et al*., 2007), *post hoc* analysis was performed using the RVAideMemoire package (Hervé, 2020). We tested whether differences in variance could have caused significant differences using permutest, which were nonsignificant for both bacterial and fungal analyses, indicating that the PERMANOVA results are valid. Differential ASVs were calculated using DESeq2 (Love *et al*., 2014), by comparing each treatment to the noninfested and noninoculated group with a false discovery rate of 0.05.

We used the packages tidyverse, lme4, emmeans, lmtest, lattice and fitdistrplus for plant and insect data (Zeileis & Hothorn, 2002; Sarkar, 2008; Bates *et al*., 2015; Delignette‐Muller & Dutang, 2015; Lenth *et al*., 2018; Wickham *et al*., 2019). The distribution of each dataset was explored with QQ‐plots, histograms, Shapiro–Wilk test and the function descdist with 2000 bootstrapped values. Analysis of leaf length, plant shoot dry biomass and gene expression levels was performed with generalized linear models either using Gamma or Gaussian distributions. Development time, fly emergence and hind tibia length of *D*. *radicum* were analyzed by using generalized linear mixed models with Poisson, binomial and gamma distributions, respectively. Plant ID was used as a random factor to avoid pseudoreplication. Models were compared and chosen based on Akaike Information Criterion (AIC) values. In the case of multiple fixed factors, the best model that included both factors (‘soil treatment’ and ‘sex’ or ‘time’) was chosen. Significance of fixed factors was assessed using the lrtest function.

## Results

### Insect herbivore‐induced alterations in the plant rhizosphere microbiome

Rhizospheres from plants in the conditioning phase were extracted and analyzed for bacterial and fungal communities. We found 1311 bacterial and 187 fungal ASVs, the majority of which belong to the phyla Proteobacteria and Ascomycota, respectively (Fig. S2).

**Fig. 2:**
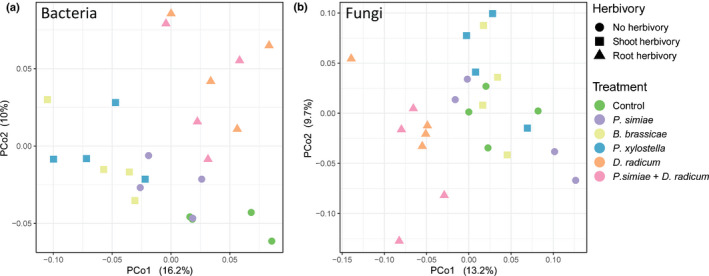
Principal coordinate analysis (PCoA) of bacterial (a) and fungal (b) rhizosphere communities. *Brassica oleracea* plants were infested with *Brevicoryne brassicae*, *Plutella xylostella* or *Delia radicum*, inoculated with *Pseudomonas simiae* WCS417r, or infested with *D. radicum* and inoculated with *Pseudomonas simiae*. Control plants were noninfested and noninoculated. After 2 wk, rhizosphere samples were collected and pooled from six plants. Bacterial 16S and fungal ITS2 regions were sequenced. Colors distinguish no herbivory, shoot or root herbivory; treatments are represented by shapes.

Multivariate analysis revealed that microbial communities clustered by the presence and feeding location of inducing herbivores (Fig. 2; Table 1). The bacterial communities in rhizospheres of plants induced by root‐feeding *D. radicum* clustered separately from those of plants induced by the shoot‐feeding insects *Brevicoryne brassicae* and *Plutella xylostella* and no herbivory (hereafter root herbivory, shoot herbivory, and no herbivory). These differences were confirmed by PERMANOVA (Table 1), which showed that these three groups indeed differ in their bacterial communities (no herbivory – shoot herbivory: *F* = 2.77, *P* < 0.001, no herbivory – root herbivory: *F* = 2.03, *P* < 0.001, shoot herbivory – root herbivory: *F* = 3.20, *P* < 0.001). Within these three groups, treatments did not differ from each other (Control – *Pseudomonas simiae*: *F* = 1.17, *P* = 0.33, *Brevicoryne brassicae* – *Plutella xylostella*: *F* = 0.84, *P* = 0.89; *D. radicum* – *Pseudomonas* *simiae* + *D. radicum*: *F* = 1.04, *P* = 0.37). Fungi were also affected by the treatments, rhizosphere fungal communities from plants treated with root herbivory separated from the other samples on the first principal component (Fig. 2; Table 1). Rhizosphere fungal communities were strongly affected by root herbivory, and only slightly by shoot herbivory (no herbivory – shoot herbivory: *F* = 1.47, *P* = 0.01; no herbivory – root herbivory: *F* = 2.34, *P* < 0.001; shoot herbivory – root herbivory: *F* = 2.48, *P* < 0.001). No differences were observed within the groups of shoot herbivory, root herbivory, or no herbivory (Control – *Pseudomonas simiae*: *F* = 0.99, *P* = 0.64; *Brevicoryne brassicae* – *Plutella xylostella*: *F* = 0.86, *P* = 0.77; *D. radicum* – *Pseudomonas* *simiae* + *D. radicum*: *F* = 1.42, *P* = 0.09). Thus, feeding on either shoot or root tissue by herbivores appears to be an important factor in shaping the rhizosphere microbial community.

**Table 1:** Effects of treatment and herbivory on bacterial and fungal communities, where herbivory consisted of six treatments; *Brassica oleracea* plants were infested with *Brevicoryne brassicae*, *Plutella xylostella*, *Delia radicum*, inoculated with *Pseudomonas simiae* WCS417r, infested with *D. radicum* and inoculated with *Pseudomonas simiae*, or noninfested/noninoculated plants used as control. These treatments were grouped into shoot, root, or no herbivory to form the herbivory factor.

Variable	Model type	Model	*F*	*R* ^2^	*P*‐value
Bacterial communities	PERMANOVA	Treatment	1.68	0.32	< 0.001
		Herbivory	2.65	0.20	< 0.001
Fungal communities	PERMANOVA	Treatment	1.49	0.29	< 0.001
		Herbivory	2.09	0.17	< 0.001

**Fig. 3:**
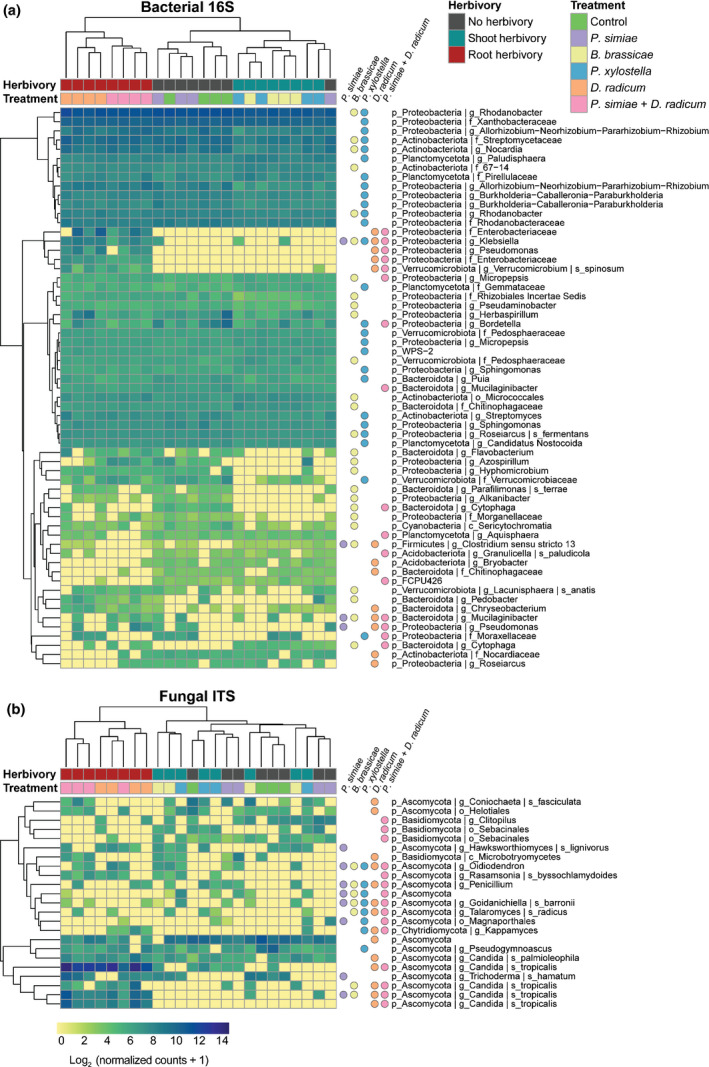
Biclustered heatmaps showing differentially abundant bacterial (a) and fungal (b) amplicon sequence variants (ASVs). *Brassica oleracea* plants were infested with *Brevicoryne brassicae*, *Plutella xylostella* or *Delia radicum*, inoculated with *Pseudomonas simiae* WCS417r, or infested with *D. radicum* and inoculated with *Pseudomonas simiae*. Control plants were noninfested and noninoculated. After 2 wk, rhizospheres were collected in four samples, each pooled from six plants. Bacterial 16S and fungal ITS2 regions were sequenced. Differentially abundant ASVs were selected by deseq2, with a threshold of false discovery rate < 0.05 difference between treatment and control. Colored circles right of the heatmaps show whether the abundance of the ASV is significantly different between that treatment and control. Clustering by shoot and root herbivory and treatment is based on Euclidean distance. Colors show log_2_(normalized count +1).

To identify specific changes caused by our treatments, we analyzed differentially abundant ASVs (Fig. 3). Based on visual representation of the Euclidean distance hierarchical tree, for both bacteria and fungi, rhizospheres of plants treated with root herbivory were separated from the shoot herbivory and no herbivory groups. Rhizospheres of plants treated with shoot herbivores also clustered in terms of bacteria, but not for fungal ASVs. For bacteria, most ASVs were differentially abundant between rhizospheres of plants treated with *Brevicoryne* *brassicae* and *Plutella xylostella* and control plants. For fungi, the largest numbers of ASVs were found for plants infested by *D. radicum* and *Pseudomonas simiae* + *D. radicum*.

A cluster of five bacterial ASVs is present in rhizospheres of plants treated with root herbivory, while being absent in the control treatment; these include two members of the family Enterobacteriaceae, a *Klebsiella*, a *Pseudomonas*, and *Verruccomicrobiom spinosum*. Among the fungal ASVs, *Candida tropicalis* has the most striking difference between treatments, and was strongly associated with rhizospheres of plants treated with *D. radicum*. Several differentially abundant ASVs, both bacteria and fungi, were negatively affected by infestation of the plants by *D. radicum* (without *Pseudomonas* *simiae*); these ASVs are members of the bacterial families Nocardiaceae and Chitinophagaceae, genera *Bryobacter*, *Chryseobacterium* and *Roseiarcus*, and fungal order Helotiales, class Microbotryomycetes, and species *Candida palmioleophila* and *Coniochaeta fasciculata*.

Further, a group of highly abundant bacterial ASVs were quantitatively affected in the rhizospheres of *Plutella xylostella* and *Brevicoryne brassicae*‐treated plants compared to control plants. For instance, a member of the genus *Rhodanobacter* was the most abundant ASV in the overall bacterial community, and it was reduced from an average of 3700 normalized counts (4.8% relative abundance) in the rhizosphere of control plants, to 2600 (3.9% relative abundance) and 2500 (3.8% relative abundance) in rhizospheres of *Plutella xylostella* and *Brevicoryne brassicae*‐treated plants, respectively. Interestingly, several bacterial ASVs were depleted specifically in rhizospheres of *Brevicoryne brassicae*‐treated plants compared to rhizosphere of control plants, including members of the genera *Flavobacterium*, *Azospirillum*, *Hyphomicrobium*, *Alkanibacter*, *Cytophaga*, and the species *Parafilimonas terrae*.

Rhizospheres of plants inoculated with *Pseudomonas simiae* only differed from those of noninfested/noninoculated plants in four bacterial ASVs, while eight fungal ASVs were affected. Of those four bacterial ASVs in rhizospheres of *Pseudomonas simiae*‐inoculated plants, one is a *Pseudomonas* fully matching *Pseudomonas simiae* WCS417r through a Blast search. However, the sequenced 16S fragments are identical to many strains in the related group Pseudomonads. Therefore we cannot verify that these fragments are explicitly from the strain used in the experiment; without specific bacterial testing, we cannot be certain of the origin of our recovered ASV. Two fungal ASVs, *Hawksworthiomyces lignovirorous* and *Trichoderma hamatum*, are specifically depleted in rhizospheres of plants inoculated with *Pseudomonas simiae*.

### Plant–soil feedback effects on plant performance

**Fig. 4:**
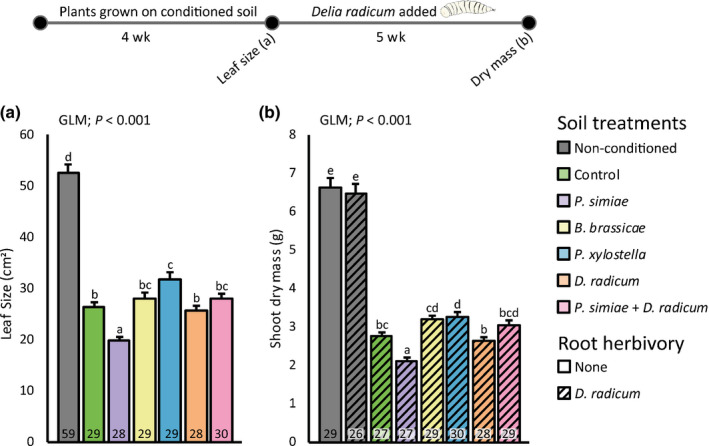
Size of leaf number 2 counted from the bottom on the stem (a) and dry shoot biomass after *Delia radicum* infestation (b) of *Brassica oleracea* plants grown in soil conditioned by conspecific plants exposed to herbivory, rhizobacterial inoculation or a combination. In the conditioning phase, *Brassica oleracea* plants were infested with *Brevicoryne brassicae*, *Plutella xylostella* or *D. radicum*, or inoculated with *Pseudomonas simiae* WCS417r, or infested with *D. radicum* and inoculated with *Pseudomonas simiae*. Control plants were noninfested and noninoculated. Plants were removed and the same soil was used to grow new *Brassica oleracea* plants. After 3 wk of growth, leaf size of these new plants was quantified before infestation with insect herbivores (a). After 5 wk of infestation, the plants were harvested, and dry shoot biomass was measured (b). All plants in the feedback phase, and a subset of plants on nonconditioned soil, were induced with 10 *D. radicum* larvae after 3 wk of growth. Numbers in bars represent the number of included plants, bars with different letters within a panel are significantly different from one another (Tukey’s honestly significant difference, *α* = 0.05), and bars show mean + SE. GLM: Generalized Linear Model.

To assess whether rhizosphere microbiome alterations affected consecutively growing plants and their resistance to insect herbivores, *Brassica* *oleracea* plants were grown in the same soil previously conditioned by conspecific plants exposed to different treatments. The surface area of the second leaf was affected by soil conditioning (Fig. 4a; Table 2): plants grown on conditioned soil had smaller leaves. Plant shoot dry mass was also affected by soil conditioning (Fig. 4b; Table 2), where dry shoot biomass of plants grown on conditioned soil was lower compared to plants grown on nonconditioned soils. Plants grown on soil conditioned by plants inoculated with *Pseudomonas simiae* were smaller compared to plants grown on soil conditioned by noninfested/noninoculated plants. Plants grown on soil conditioned by plants treated with *Plutella xylostella* were larger, both in terms of leaf size and biomass.

**Table 2:** Effects of the factors soil treatment, root herbivory and sex on *Delia radicum* performance variables, and effects on plant performance and gene expression of *Brassica oleracea*.

Variable	Model type	Model	Factor	χ^2^	df	*P*‐value
*Delia radicum*	GLMM	Soil treatment + PlantID^a^	Soil treatment	25.62	6	< 0.001
emergence	Binomial					
*Delia radicum*	LMM	Soil treatment + Sex + PlantID^a^	Soil treatment	14.18	6	0.028
tibia length	Normal		Sex	68.87	1	< 0.001
*Delia radicum*	GLM	Soil treatment	Soil treatment	15.56	6	0.016
18S	Gamma					
Leaf area	GLM	Soil treatment	Soil treatment	383.57	6	< 0.001
	Gamma					
Plant dry mass	GLM	Soil treatment	Soil treatment	336.44	7	< 0.001
	Gamma					
Root *LOX6*	GLM	Soil treatment + Root herbivory	Soil treatment	6.13	6	0.408
	Gamma		Root herbivory	33.27	1	< 0.001
Root *MYB28*	GLM	Soil treatment × Root herbivory	Soil treatment	1.82	6	0.935
	Gamma		Root herbivory	125.31	1	< 0.001
			Interaction	27.84	6	< 0.001
Root *CYP81F4*	GLM	Soil treatment × Root herbivory	Soil treatment	1.33	6	0.97
	Gamma		Root herbivory	105.76	1	< 0.001
			Interaction	15.09	6	0.02
Root *MYB72*	GLM	Soil treatment + Root herbivory	Soil treatment	20.27	6	0.002
	Gamma		Root herbivory	0.57	1	0.451
Root *PDR9*	GLM	Soil treatment × Root herbivory	Soil treatment	31.83	6	< 0.001
	Gamma		Root herbivory	20.91	1	< 0.001
			Interaction	23.71	6	< 0.001
Leaf *LOX2*	LM	Soil treatment × Root herbivory	Soil treatment	6.75	6	0.344
	Normal		Root herbivory	37.86	1	< 0.001
			Interaction	14.91	6	0.021
Leaf *MYB28*	GLM	Soil treatment × Root herbivory	Soil treatment	30.26	6	< 0.001
	Gamma		Root herbivory	3.46	1	0.063
			Interaction	8.98	6	0.175

(G)L(M)M, (Generalized) Linear (Mixed) Model.

^a^PlantID was included in the models as a random factor to avoid pseudoreplication as multiple flies emerged from each plant.

### Plant–soil feedback effects on *D. radicum* performance

To examine belowground plant resistance in a PSF context, we infested *Brassica* *oleracea* plants grown in conditioned soils with *D*. *radicum* larvae. Overall, *D. radicum* adult emergence was low in the experiment, on average 11.4% (*N*_total_ = 1970) of larvae developed into adults. In addition to these performance measurements, in the plants used for gene expression analysis, we examined larval performance through analysis of *D. radicum* 18S ribosomal RNA.

**Fig. 5:**
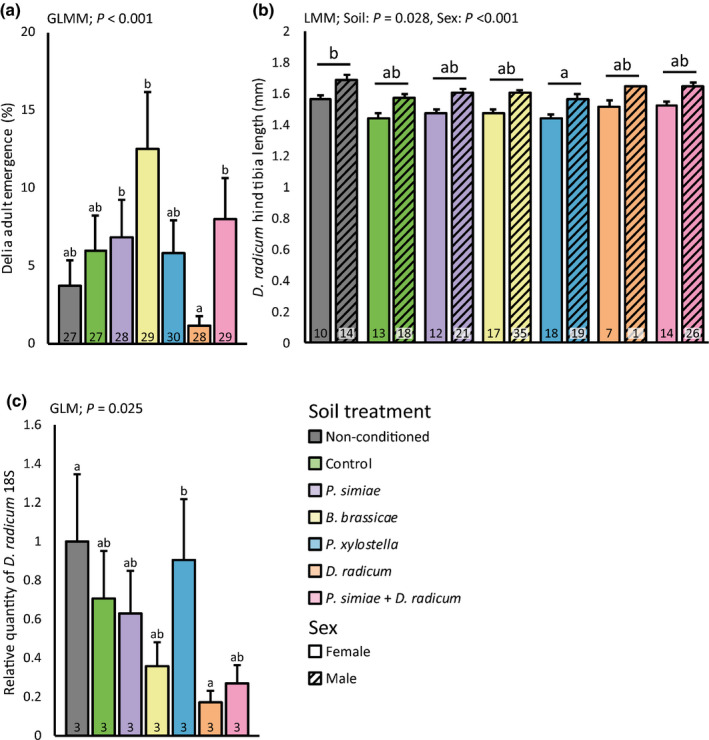
*Delia radicum* adult emergence (a), hind tibia length (b), and relative quantity of *D*. *radicum* 18S rRNA (c) in the primary roots of *Brassica oleracea* plants grown in soil conditioned by conspecific plants exposed to herbivory, rhizobacterial inoculation or a combination. In the conditioning phase, *Brassica oleracea* plants were infested with *Brevicoryne brassicae*, *Plutella xylostella* or *D. radicum*, or inoculated with *Pseudomonas simiae* WCS417r, or infested with *D. radicum* and inoculated with *Pseudomonas simiae*. Control plants were noninfested and noninoculated. Plants were removed and the same soil was used to grow new *Brassica oleracea* plants. After 3 wk of growth, these new plants were infested with *D. radicum* larvae, emerging flies counted and their hind tibia length measured, and in separate experimental plants the amount of *D*. *radicum* 18S was assessed 24 h after infestation. Numbers in bars represent the number of plants (a) flies (b), or pools of four plants (c), bars with different letters are significantly different from each other (Tukey’s honestly significant difference, *α* = 0.05), and bars show mean + SE. Due to low sample size, no SE could be calculated for males in the *D. radicum* treatment (orange striped bar). Soil, Soil conditioning treatment; (G)LMM, (Generalized) Linear Mixed Model.

Emergence of *D*. *radicum* was affected by soil conditioning in a treatment‐specific way (Fig. 5a; Table 2). Fewer flies emerged from plants grown on soil conditioned by plants infested by *D. radicum* compared to plants grown on soils conditioned by plants treated with *Brevicoryne brassicae, Pseudomonas simiae* or *D. radicum* together with *Pseudomonas simiae*. Tibia length of adult flies was affected by soil conditioning (Fig. 5b; Table 2). Flies with smaller tibia length emerged from plants grown on soil conditioned by plants infested with *Plutella xylostella* compared to flies that emerged from plants grown on nonconditioned soil. Fly development time was similar for all treatments (data not shown).

In the set of plants used for gene expression analysis 24 h post‐infestation, we quantified *D. radicum* 18S ribosomal RNA relative to plant reference genes as a proxy of *D. radicum* performance (Fig. 5c; Table 2). Relative quantities of *D. radicum* 18S were affected by soil‐conditioning treatments. This analysis supports the observation that *D*. *radicum* performance was reduced in plants grown on soil conditioned by *D. radicum* compared to plants grown on nonconditioned soil or soil conditioned by control plants. Taken together, the results show that *D. radicum* was negatively affected when feeding on plants that had been growing in soil conditioned by plants also exposed to feeding by conspecific larvae.

### Gene expression in response to *D. radicum* infestation and plant–soil feedback treatments

**Fig. 6:**
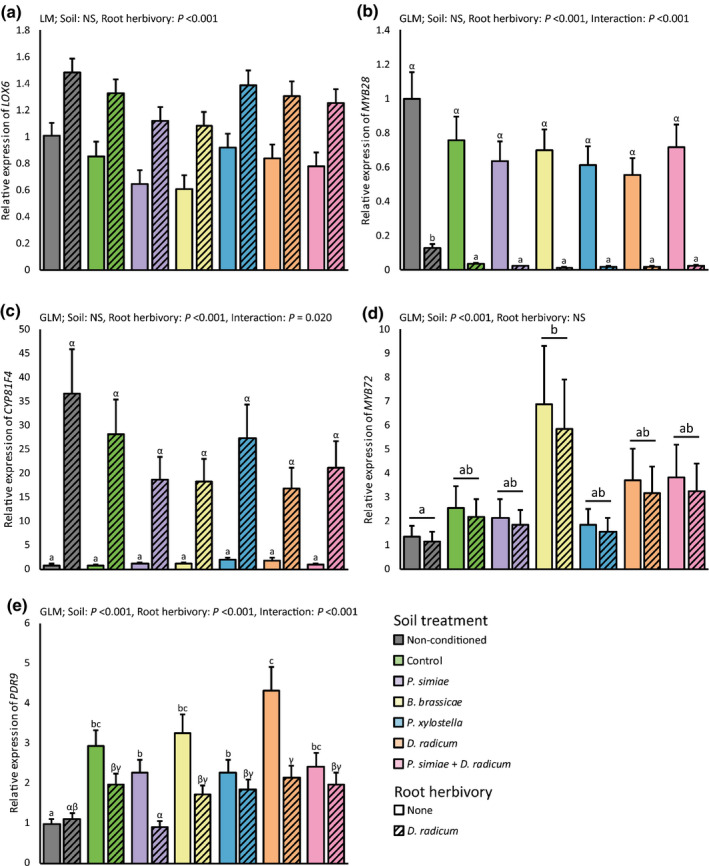
Relative gene expression of *LOX6* (a), *MYB28* (b), *CYP81F4* (c), *MYB72* (d), and *PDR9* (e) in the primary roots of *Brassica oleracea* plants grown in soil conditioned by conspecific plants exposed to herbivory, rhizobacterial inoculation or a combination. In the conditioning phase, *Brassica oleracea* plants were infested with *Brevicoryne brassicae*, *Plutella xylostella* or *Delia radicum*, or inoculated with *Pseudomonas simiae* WCS417r, or infested with *D. radicum* and inoculated with *Pseudomonas simiae*. Control plants were noninfested and noninoculated. Plants were removed and the same soil was used to grow new *Brassica oleracea* plants. After 3 wk of growth, half of these plants were infested with *D. radicum* (striped bars). All bars are set relative to the gene expression levels in primary roots of plants of noninfested plants grown in nonconditioned soil (gray bar). Bars with different letters are significantly different from one another, within *D*. *radicum* infested plants (Greek alphabet) or plants that did not receive root herbivores (Roman alphabet; Tukey’s honestly significant difference, *α* = 0.05), and bars show mean + SE. Soil, Soil conditioning treatment; NS, not significant; (G)LM, (Generalized) Linear Model; *n* = three or four replicates of three pooled plants.

We assessed primary root defense responses to herbivory by *D*. *radicum* in plants grown on conditioned and nonconditioned soil, measured after 24 h of *D*. *radicum* infestation of the primary root. Expression in the roots of *LOX6*, a gene involved in JA biosynthesis, was induced by *D*. *radicum* regardless of soil conditioning (Fig. 6a; Table 2). Root transcript levels of *MYB28*, involved in the biosynthesis of aliphatic glucosinolates, were downregulated by *D*. *radicum* infestation (Fig. 6b; Table 2). The soil conditioning treatments did not affect root *MYB28* expression, but there was a significant interaction effect between *D*. *radicum* infestation and soil conditioning. When infested with *D. radicum*, transcript levels of *MYB28* were lower in plants grown on conditioned soils compared to nonconditioned. In contrast to *MYB28* downregulation by *D*. *radicum* infestation, messenger RNA (mRNA) levels of *CYP81F4*, encoding an enzyme involved in indole glucosinolates biosynthesis, were strongly upregulated by infestation. Type of soil conditioning did not influence *CYP81F4* transcript levels, but there was an interaction between *D*. *radicum* and soil conditioning (Fig. 6c; Table 2).

Expression of root *MYB72*, a transcription factor involved in induced systemic resistance and iron acquisition (van der Ent *et al*., 2008; Palmer *et al*., 2013), was affected by soil conditioning in a treatment‐specific way, but not by *D*. *radicum* infestation (Fig. 6d; Table 2). Transcript levels of *PDR9*, a gene encoding a transporter involved in root exudation of coumarins, were affected by both soil treatment and *D*. *radicum* infestation, and there was an interaction between soil treatment and *D. radicum* infestation (Fig. 6e; Table 2). When no *D*. *radicum* was present, expression of *PDR9* was upregulated in primary roots of plants subjected to all soil conditioning treatments compared to plants grown on nonconditioned soil, especially when soil was conditioned by plants infested with *D*. *radicum*. This effect was attenuated upon *D*. *radicum* infestation, in which case transcript levels of *PDR9* did not differ between soil conditioning treatments.

Leaf transcript levels of *LOX2*, a marker gene for JA biosynthesis expressed in the shoot, were increased by root herbivory but not by soil conditioning; there was a significant interaction effect between soil conditioning and root herbivory (Fig. S3a; Table 2). *MYB28* transcript levels in leaves were affected by soil conditioning treatments (Fig. S3b; Table 2), but not by *D. radicum* infestation.

## Discussion

Our study shows that the plant root microbiome is affected by insect attack to the plant and that plant growth and insect resistance are influenced via PSF mechanisms. Our results demonstrate that the bacterial rhizosphere community is differentially affected by shoot and root herbivory, whereas the fungal rhizosphere community is mostly affected by root herbivory. Although previous research shows that plant defense against shoot‐feeding insects can be altered through PSF (Kostenko *et al*., 2012; Bezemer *et al*., 2013; Kos *et al*., 2015a,b; Hu *et al*., 2018; Pineda *et al*., 2020), we here show novel evidence that the root‐feeding insect *D. radicum* is negatively affected by conspecific feeding through PSF. While our data do not allow an unambiguous link to be established between the rhizosphere microbiome in the conditioning phase and the results in the feedback phase, it is most plausible that microbial changes underlie the reported PSF effects on plant growth and insect resistance.

### Rhizosphere microbiome composition is differentially affected by shoot and root herbivory

We observed that herbivores feeding on the root or the shoot influenced the rhizosphere microbial community. Multivariate analysis revealed that bacterial rhizosphere communities were separated into three groups: (1) plants exposed to shoot herbivory, (2) plants exposed to root herbivory and (3) noninfested plants. We further observed that the fungal rhizosphere community was similar between plants fed on by shoot‐feeding insects and noninfested plants, but was different from the fungal community of plants with root‐feeding *D. radicum*. Thus, our results show that root herbivory has more impact on the plant rhizosphere community than the addition of *Pseudomonas simiae* WCS417r. A previous study showed that *D. radicum* herbivory led to only minor changes in the fungal community, but caused major changes in both endosphere and rhizosphere bacterial communities of oilseed rape, *Brassica napus* (Ourry *et al*., 2018). Interestingly, our results show that *D. radicum* herbivory strongly increased the abundance of the soil yeast *Candida tropicalis*, a species containing known plant growth promoting strains (Amprayn *et al*., 2012). None of the fungal ASVs that were different between the treatments are known to have entomopathogenic properties, although this was not directly studied for most of these species. Rhizospheres of *D. radicum*‐infested plants showed an accumulation of several bacterial taxa (Enterobacteriaceae, *Klebsiella*, and *Pseudomonas*) that were previously found to be associated with the *D. radicum* gut microbiome (Lukwinski *et al*., 2006; van den Bosch & Welte, 2020). The gut microbiome of another much‐studied root herbivore, western corn rootworm, is thought to consist mostly of microbes selected from the surrounding soil (Dematheis *et al*., 2012; Ludwick *et al*., 2019). Our findings hint at the interesting possibility of direct interactions between the microbiomes of the plant rhizosphere and the root herbivore gut. Perhaps, by selecting specific microbes from the soil and excreting them, root herbivores can influence the rhizosphere microbiome.

Herbivory by shoot‐feeding insects was previously shown to affect the rhizosphere community, in line with our results (Yang *et al*., 2011; Lee *et al*., 2012; Bezemer *et al*., 2013; Kong *et al*., 2016; Malacrinò *et al*., 2020; Zytynska *et al*., 2020). However, some studies report similar rhizosphere microbiomes between shoot–herbivore‐infested and noninfested plants (O'Brien *et al*., 2018; Malacrinò *et al*., 2020). The variation seen in the literature regarding rhizosphere microbiome responses to shoot herbivory could be explained by factors such as plant‐ and insect‐specific responses, or different bulk soil bacterial communities in the starting soil.

### Plant–soil feedback by differently treated conspecifics has adverse effects on plant growth

In the feedback phase of our experiment, we observed treatment‐dependent responses in plant growth when grown on conditioned soils. Regardless of the treatment, plant growth was inhibited on conditioned soil compared to nonconditioned soil. Generally, such unfavorable legacy from plant conspecifics is termed negative PSF. In our experiment, shoot herbivory by *Plutella xylostella* on plants during the conditioning phase led to increased growth of plants in the feedback phase, compared to plants grown in soil conditioned by plants without herbivores. Hence, herbivory can affect not only the attacked plant, but also the growth of future plants growing in the same soil, via soil‐mediated effects.

It is challenging to directly link changes in the rhizosphere microbiome of plants in the conditioning phase of our experiment with findings in the feedback phase. One potential discrepancy is that we sampled rhizosphere soil for microbiome analysis but transferred all soil in the pot to the feedback phase. The soil in the pots was completely colonized by roots at the end of the conditioning phase, therefore we believe that the overall bacterial community we transferred is representative of the rhizosphere community. Several PSF mechanisms other than the transfer of microbes could have contributed to our results. Fresh litter, such as fine roots, can stimulate the microbial activity (Fontaine *et al*. 2003), but can also negatively affect plant growth through the release of phytotoxic (allelopathic) and autotoxic compounds when decomposing (Bonanomi *et al*., 2006). Extracellular self‐DNA (eDNA) is also released from decomposing tissue, and can exert plant growth inhibition on grasses, forbs and *Arabidopsis thaliana in vitro* (Mazzoleni *et al*., 2015). These PSF mechanisms are likely to have contributed to our results to some extent, as root fragments were present in the soil we transferred.

Surprisingly, the performance of *Brassica oleracea* was drastically decreased when grown in soil on which previously growing plants had been inoculated with *Pseudomonas simiae*, compared to the other soil conditioning treatments. Root herbivory by *D. radicum* together with *Pseudomonas simiae* inoculation of the plants during the conditioning phase restored plant biomass to a certain degree in the feedback phase. Although this PGPR strain is usually considered a beneficial rhizobacterium when applied to plants, including *Brassica* *oleracea* (Friman *et al*., 2021a), our results suggest that this beneficial effect may not persist through PSF. Notably, there are reports of rhizobacteria causing effects varying from plant‐growth promotion to inhibition, depending on e.g. phosphate availability or rhizobacterial population density (Ciccillo *et al*., 2002; Morcillo *et al*., 2020). Although plant growth may have been boosted in 2 wk of the conditioning phase, we regard this period as too short to leave lasting nutrient deficiencies in the soil, and therefore unlikely to have influenced our results. Further, we assume that the nutrient availability was sufficient for the experimental plants due to regular fertilization in our experiments and hypothesize that changes in the microbiome underlie the reduction in growth.

In contrast to our hypothesis, we found that inoculation with the rhizobacterium *Pseudomonas* *simiae* did not affect overall microbial communities in the rhizosphere. Although there are studies that find an altered root community after addition of individual rhizobacterial species, others report no such effects (Herschkovitz *et al*., 2005; Gadhave *et al*., 2018; Wang *et al*., 2018; Zytynska *et al*., 2020). Even though the microbial community composition was not affected by the addition of *Pseudomonas simiae* WCS417, the abundance of several distinct species was changed. It has been demonstrated that only a set of three bacterial soil species are sufficient to increase resistance in *Arabidopsis thaliana* against a foliar fungal pathogen (Berendsen *et al*., 2018). For example, *Trichoderma hamatum* was absent in rhizospheres of *Pseudomonas simiae‐*induced plants while it was present in the other treatments. This species is a known growth‐promoting fungal species in e.g. pepper (Mao *et al*., 2020). In this way, the addition of *Pseudomonas simiae* may have suppressed other beneficial microbes in the rhizosphere, leading to a net negative effect on plant growth in our study.

### Root herbivores can be affected via plant–soil feedback

Root herbivory by *D. radicum* during the conditioning phase led to lower performance of *D. radicum* in the feedback phase, in line with previous studies that recorded an alteration of plant resistance against insects through PSF (Kostenko *et al*., 2012; Bezemer *et al*., 2013; Kos *et al*., 2015a,b; Hu *et al*., 2018; Pineda *et al*., 2020). Overall *D. radicum* adult emergence in our experiment was low compared to other studies using similar methods (Soler *et al*., 2007; van Geem *et al*., 2015; Karssemeijer *et al*., 2020). As a root miner, the insect is difficult to quantify in the early stages of its lifecycle. Therefore, we developed primers to supplement the emergence data with the quantification of *D. radicum* 18S ribosomal RNA after 24 h of feeding. This is a novel method to quantify root fly larval performance *in planta*; yet, similar methods are used to quantify plant parasitic nematode abundance in roots (Zijlstra & Van Hoof, 2006; Braun‐Kiewnick *et al*., 2016). The *D. radicum* 18S ribosomal RNA method confirmed a lower performance of *D. radicum* on plants in the feedback phase growing in soil conditioned with *D. radicum*‐infested plants. Notably, this technique can be further fine‐tuned, for instance by dilution or selecting the optimal time‐point for harvesting, and the results here should be interpreted in conjunction with the emergence data. Differences between the emergence data and 18S measurements may be due to different life stages targeted, as one measures performance of neonates while the other measures survival to adulthood.

The performance of *D. radicum* may have been affected by a change in plant defense, or by a direct influence of the soil microbiome. Lachaise *et al*. (2017) reported that differences in the soil microbiome affected *D. radicum* performance. *Delia radicum* infestation was previously shown to increase the abundance of *Bacillus* and *Paenibacillus* in the rhizosphere, which could have entomopathogenic properties (Ourry *et al*., 2018). These bacterial species were not differentially affected in our study, perhaps due to different plant growth substrates. Without isolating specific rhizosphere microbes and testing their effects on the plant and the root herbivore larvae, we can only speculate about the underlying mechanisms.

In roots, most defense markers we studied were not affected by soil conditioning treatments, and thus they do not explain the difference in insect performance. However, we cannot rule out that soil microbes may have primed defense against *D. radicum*, leading to a faster defensive response. Indeed, two genes involved in ISR, *MYB72* and *PDR9*, were affected by soil conditioning treatments. The role of these genes in ISR has been especially studied in *Arabidopsis thaliana*. Here, we found that soil conditioning changed the expression of their orthologues in *Brassica oleracea*. The transcription factor MYB72 has been identified as a key regulation node in *Arabidopsis thaliana* roots in iron uptake and communication with the beneficial rhizobacterium *Pseudomonas* *simiae* WCS417r (Verhagen *et al*., 2004) and was later verified to play a central role in rhizobacterial ISR (van der Ent *et al*., 2008). This transcription factor regulates the expression of genes involved in the shikimate, phenylpropanoid and nicotianamine biosynthesis pathways, including genes leading to the production and exudation of coumarins (Zamioudis *et al*., 2014). These coumarins, in particular scopoletin, are secreted by the roots by the transporter *PDR9*, where they play a dual role in both the plant response to iron deficiency and influencing the rhizosphere microbiome (Stringlis *et al*., 2018, 2019). This could be an indication that ISR plays a role in PSF. Interestingly, transcript levels of *LOX2* and *MYB28* in leaves were affected by soil conditioning treatments, a result which is in line with previous studies that found a link between shoot defense and PSF in maize plants (Hu *et al*., 2018). Our gene expression results underline that defense signaling in shoot and root is fundamentally different (Johnson *et al*., 2016).

### Conclusion

In conclusion, our study demonstrates that shoot and root herbivory lead to distinct plant rhizosphere microbial communities, whereas inoculation of *Pseudomonas simiae* to the soil has limited effects on the rhizosphere microbial community. Through PSF, plant performance and defense is altered in a treatment‐dependent way for *Brassica oleracea* plants growing in soil conditioned by conspecific plants. The results presented here suggest that changes in the abundance of specific microbes, rather than the overall microbiome, may be more important for plant performance and defense.

## Author contributions

PNK and JF designed the study. JH together with JF and PNK conducted the experiments and performed molecular analysis. JH, JF and PNK analysed the data with assistance of JJAvL and MD. KdK and PNK developed the *Delia radicum* 18S primers. JF and PNK wrote the manuscript, with the help of JJAvL and MD. All authors approved the final version of the manuscript. JF, PNK and JH share first authorship of this work.

## Supporting information


**Fig. S1** Relative quantity of *Delia radicum* 18S in different life stages of *D. radicum* and in plants infested with different numbers of neonate larvae.
**Fig. S2** Relative abundance of bacterial and fungal phyla in rhizospheres of *Brassica oleracea* plants exposed to herbivory, rhizobacterial inoculation or a combination.
**Fig. S3** Relative gene expression of *LOX2* and *MYB28* in leaves of *Brassica oleracea* plants grown in soil conditioned by conspecific plants exposed to herbivory, rhizobacterial inoculation or a combination.
**Methods S1** *Delia radicum* biomass assessment.
**Notes S1** Code used for processing and analyzing microbiome samples.
**Table S1** Primers for target and reference genes in *Brassica oleracea*.
**Table S2** *Delia radicum* specific primer pairs.
Please note: Wiley Blackwell are not responsible for the content or functionality of any Supporting Information supplied by the authors. Any queries (other than missing material) should be directed to the *New Phytologist* Central Office.

## Data Availability

The data that support the findings of this study are available from the corresponding author upon reasonable request. Sequencing data are available on https://www.ebi.ac.uk/ena/ with study accession no. PRJEB47452.
