# Supplementary material for: Shoot and root insect herbivory change the plant rhizosphere microbiome and affects cabbage–insect interactions through plant–soil feedback
Source: New Phytol. 2021 Oct 9;232(6):2475–90. doi: 10.1111/nph.17746 (PMC9291931; doi:10.1111/nph.17746)
Supplement: Supplementary file 1 — Fig. S1 Relative quantity of Delia radicum 18S in different life stages of D. radicum and in plants infested with different numbers of neonate larvae. Fig. S2 Relative abundance of bacterial and fungal phyla in rhizospheres of Brassica oleracea plants exposed to herbivory, rhizobacterial inoculation or a combination. Fig. S3 Relative gene expression of LOX2 and MYB28 in leaves of Brassica oleracea plants grown in soil conditioned by conspecific plants exposed to herbivory, rhizobacterial inoculation or a combination. Methods S1 Delia radicum biomass assessment. Notes S1 Code used for processing and analyzing microbiome samples. Table S1 Primers for target and reference genes in Brassica oleracea. Table S2 Delia radicum specific primer pairs. Please note: Wiley Blackwell are not responsible for the content or functionality of any Supporting Information supplied by the authors. Any queries (other than missing material) should be directed to the New Phytologist Central Office. [file NPH-232-2475-s001.pdf]

**_New Phytologist_ Supporting Information**

Article title: Shoot and root insect herbivory change the plant rhizosphere microbiome and affects cabbage-insect interactions through plant-soil feedback

Authors: Julia Friman, Peter N. Karssemeijer, Julian Haller, Kris de Kreek, Joop J.A. van Loon, Marcel Dicke

Article acceptance date: 09 September 2021

The following Supporting Information is available for this article:

**Table S1** Primers for target and reference genes in *Brassica oleracea.*

**Methods S1** *Delia radicum* biomass assessment.

**Table S2** *Delia radicum* specific primer pairs.

**Fig. S1** Relative quantity of *D. radicum* 18S in different life stages of *D. radicum* and in plants infested with different numbers of neonate larvae.

**Fig. S2** Relative abundance of bacterial and fungal phyla in rhizospheres of *Brassica oleracea* plants exposed to herbivory, rhizobacterial inoculation or a combination.

**Fig. S3** Relative gene expression of *LOX2* and *MYB28* in leaves of *Brassica oleracea* plants grown in soil conditioned by conspecific plants exposed to herbivory, rhizobacterial inoculation or a combination.

**Notes S1** Code used for processing and analysing microbiome samples

**Table S1** Primers for target and reference genes in *Brassica oleracea.*

| Gene | Forward primer ('5 to 3') | Reverse primer ('3 to 5') | Tissue |
|---|---|---|---|
| *LOX2* | GCCATTGAGTTGACTCGTCC | GGATGCATGGCACTTAGTTGT | Leaf |
| *LOX6* | AGGAGCTGCCAATTCGAAGC | CGCCTGTTCCAAAGTCATTCCA | Root |
| *CYP81F1* | TGTGTCAGAAACGTTCAGGCT | ATGGCACGTCGTATCCTCCG | Root |
| *MYB28* | CGGGAGAGATGAGCACAATACG | CAGCCCTCGAAGTTTCCTATCA | Leaf/Root |
| *MYB72* | AAACAAGTGGTCAAAGATCGCG | ACACTCATCTCAAGAAACGACT | Root |
| *PDR9* | ATTCCACCACCTTCTATGCCG | ACTTGGTTGTATCTGGCTCC | Root |

| Gene | Forward primer ('5 to 3') | Reverse primer ('3 to 5') | Tissue |
|---|---|---|---|
| *SAR1a* | ATCTCTAGCCACCGTTCCCT | TTCCTGACGATGCTGCACAT | Leaf/Root |
| *Btub* | GTCAAGTCCAGCGTCTGTGA | TCACACGCCTGAACATCTCC | Leaf/Root |
| *Act-2* | ACATTGTGCTCAGTGGTGGA | TCTGCTGGAATGTGCTGAGG | Leaf/Root |
| *PER4* | TATCCTCTGCAGCCTCCTCA | ACACACAGACTGAAGCGTCC | Leaf/Root |
| *GADPH* | GCTACGCAGAAGACAGTTGATGG | TGGGCACACGGAAGGACATAC | Leaf/Root |
| *EF1a* | GGTACCTCCCAGGCTGATTG | TCAGGTAKGAAGACACCTCCTTG | Leaf/Root |

**Methods S1** *Delia radicum* biomass assessment.

To assess performance of *D. radicum* while the larvae are still within the primary root, we developed species-specific primers. As the goal was to be able to measure low quantities of *D. radicum* within cabbage roots, RNA of the 18S and 28S ribosomal subunits was targeted. During *in silico* primer development, specificity of *D. radicum* primers was optimized by testing specifically for BLAST hits on Sciaridae, Nematoda, Fungi and *B. oleracea*; as these are hypothesized to be the most common non-target organisms in our samples (https://www.ncbi.nlm.nih.gov/tools/primer-blast/). Four primer pairs were further tested, of which one was selected (in bold) for the experiment (Table **S2**).

**Table S2** *Delia radicum* specific primer pairs.

| Gene | Forward primer ('5 to 3') | Reverse primer ('3 to 5') |
|---|---|---|
| ***18S*** | **GCAAGATCGTTATTATGGTTGAACTCT** | **GAACCCTGATTCCCCGTTACC** |
| *18S* | CCGGTGGAGTTCTTATATGTATTAGGT | ACCAATGAAAGTAGAACAGAGGTCTTAT |
| *28S* | GATAATGGTGCTTCTGTGCTATTGTC | TTGAGAGATGTACCGCCCCA |
| *28S* | GATAATGGTGCTTCTGTGCTATTGTC | CCTTGAATTGGATCATACCGGAGTA |

Stability of primers across different life stages of *D. radicum* was confirmed by testing primers on cDNA extracted from neonate larvae, 2- and 3-week-old larvae and pupae (Fig. **S3**). *Delia radicum* RNA was extracted with Isolate II Plant RNA kit (GCbiotech, the Netherlands) following the manufacturer’s instructions. RNA was converted to cDNA using cDNA Synthesis Kit (SensiFAST, Bioline). Quantitative polymerase chain reaction (qPCR) analysis was performed to test transcript levels of genes of interest (CFX96™ Real-Time System, Bio-rad, Hercules, CA, USA). Expression data was processed using qBase and data analysed in R. Gel electrophoresis and melt curves indicated no non-target products of different lengths. Expression was stable across life-stages for each primer.

To assess the ability of this novel technique to discriminate between different larval densities in planta, 3-week-old *B. oleracea* plants were induced with 2, 4, 6, 8, 10 neonate *D. radicum* larvae. After 24h, primary roots were harvested by uprooting the plants, cutting off

secondary roots, and freezing the samples directly in liquid nitrogen. Samples were pooled for three plants. Analysis was performed as described above.

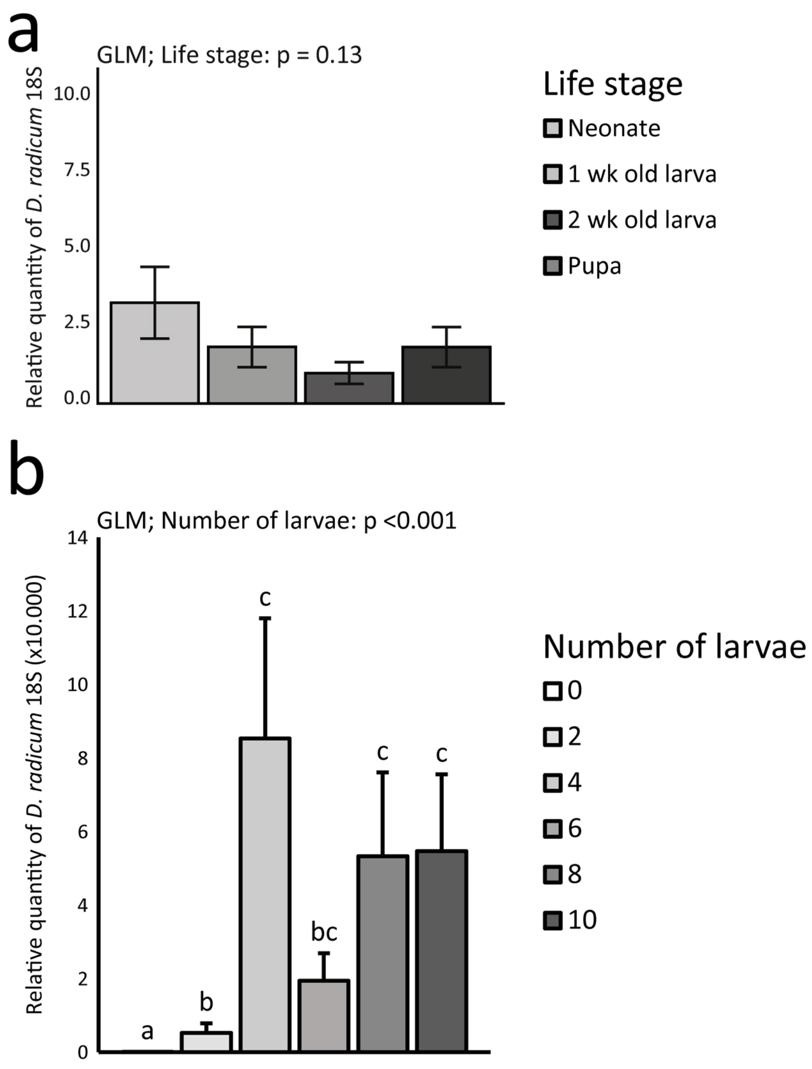


**Fig. S1** (**a**) Relative gene expression of *D. radicum* 18S in life stages of *D. radicum* and (**b**) the relative quantity of *D. radicum* 18S in plants infested with different numbers of neonate larvae. Bars show mean ± SE; bars with different letters are significantly different from one another. GLM: Generalized Linear Model. N = 4 insect individuals (**a**), or 3-5 pools of 3 plants (**b**).

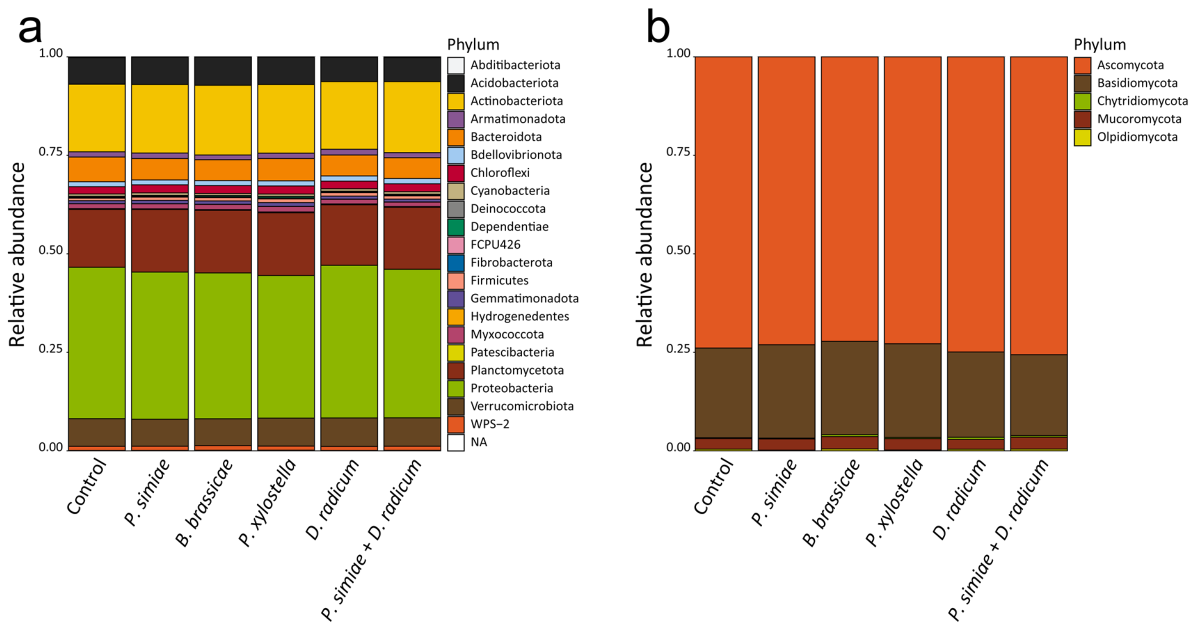


**Fig. S2** Relative abundance of bacterial (**a**) and fungal (**b**) phyla in rhizospheres of *Brassica oleracea* plants exposed to herbivory, rhizobacterial inoculation or a combination.

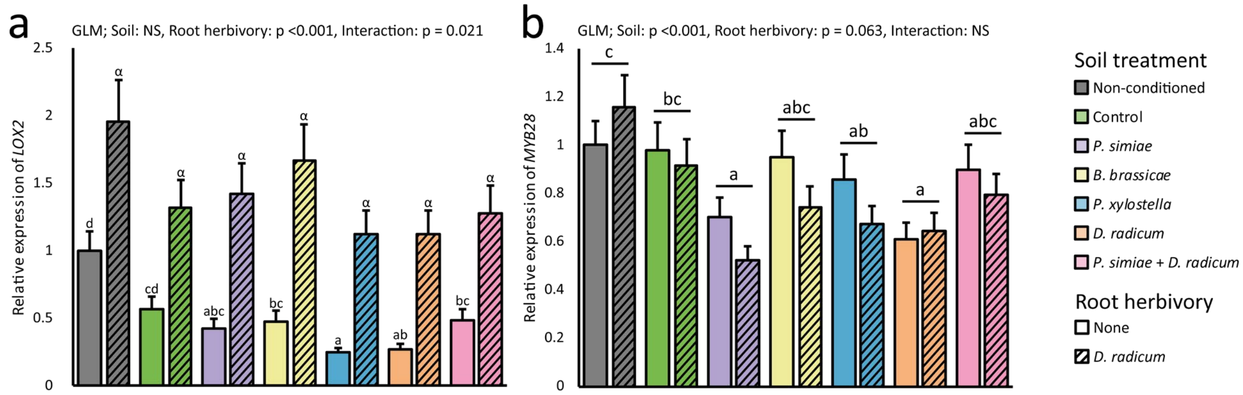


**Fig. S3** Relative gene expression of *LOX2* (**a**) and *MYB28* (**b**) in leaves of *Brassica oleracea* plants grown in soil conditioned by conspecific plants exposed to herbivory, rhizobacterial inoculation or a combination. In the conditioning phase, *B. oleracea* plants were infested with *Brevicoryne brassicae*, *Plutella xylostella* or *Delia radicum*, inoculated with *Pseudomonas simiae* WCS417, or infested with *D. radicum* and inoculated with *P. simiae*. Plants were removed and the same soil was used to grow new *B. oleracea* plants. After three weeks of growth, half of these plants were infested with *D. radicum* (x-axis). All bars are set relative to the gene expression levels in leaves from plants grown in non-conditioned soil and were non-infested (white bar). Bars show mean + SE; bars with different letters are significantly different from one another, within control (Roman alphabet) or *D. radicum* infested (Greek alphabet) samples (Tukey's HSD, α = 0.05). Soil: soil conditioning treatment; NS: Not significant, GLM: Generalized Linear Model. N = 3 or 4 replicates of pools of three plants.

**Notes S1** Code used for processing and analysing microbiome samples

The following R scripts were used for processing and analysis of bacterial 16S and fungal ITS data.

16S_dada.R (starts page 7)

ITS_dada.R (starts page 12)

16S_analysis.R (starts page 16)

ITS_analysis.R (starts page 20)

# 16S_DADA.R

```
# Shoot and root insect herbivory changes the plant rhizosphere microbiome and affects cabbage-insect interactions through plant-soil feedback
#
# This script is used for processing raw 16S reads using DADA2
# by Julia Friman and Peter Karssemeijer
#
#
#
# https://benjjneb.github.io/dada2/tutorial.html # DADA2 tutorial we followed
#
#

rm(list=ls())                                                    ## clear workspace ##

#::install("phyloseq")
#BiocManager::install("dada2")

library(dada2); packageVersion("dada2")
library(ShortRead); packageVersion("ShortRead")
library(Biostrings); packageVersion("Biostrings")

# Allow multithreading
library(RcppParallel)
setThreadOptions(numThreads = 8)
defaultNumThreads()

# Set working directory
setwd(dir = "my/favourite/folder")

### Set file names ####

path <- "Raw_Data/16S"
list.files(path)

# Forward and reverse fastq filenames have format: SAMPLENAME_R1_001.fastq and SAMPLENAME_R2_001.fastq
fnFs <- sort(list.files(path, pattern="_R1.fastq", full.names = TRUE))
fnRs <- sort(list.files(path, pattern="_R2.fastq", full.names = TRUE))
# Extract sample names, assuming filenames have format: SAMPLENAME_XXX.fastq
sample.names <- sapply(strsplit(basename(fnFs), "_"), `[`, 1)

### Remove primers ###
FWD <- "GTGCCAGCMGCCGCGGTAA"  ## 515FW primer
REV <- "GGACTACHVGGGTWTCTAAT"  ## 806RV primer

# produce all orientations #

allOrients <- function(primer) {
  # Create all orientations of the input sequence
  require(Biostrings)
  dna <- DNAString(primer)  # The Biostrings works w/ DNAString objects rather than character vectors
  orients <- c(Forward = dna, Complement = complement(dna), Reverse = reverse(dna),
               RevComp = reverseComplement(dna))
  return(sapply(orients, toString))  # Convert back to character vector
}
FWD.orients <- allOrients(FWD)

REV.orients <- allOrients(REV)

# Filter N

fnFs.filtN <- file.path(path, "filtN", basename(fnFs)) # Put N-filterd files in filtN/ subdirectory
fnRs.filtN <- file.path(path, "filtN", basename(fnRs))
filterAndTrim(fnFs, fnFs.filtN, fnRs, fnRs.filtN,
              maxN = 0,
              rm.phix=TRUE,
              compress=FALSE,
              multithread=TRUE,
              verbose = TRUE)

# Check the presence of primers
primerHits <- function(primer, fn) {
  # Counts number of reads in which the primer is found
  nhits <- vcountPattern(primer, sread(readFastq(fn)), fixed = FALSE)
  return(sum(nhits > 0))
```

```
}


rbind(FWD.ForwardReads = sapply(FWD.orients, primerHits, fn = fnFs.filtN[[1]]),
    FWD.ReverseReads = sapply(FWD.orients, primerHits, fn = fnRs.filtN[[1]]),
    REV.ForwardReads = sapply(REV.orients, primerHits, fn = fnFs.filtN[[1]]),
    REV.ReverseReads = sapply(REV.orients, primerHits, fn = fnRs.filtN[[1]]))


### Run Cutadapt! ####

path.cut <- file.path(path, "cutadapt")
if(!dir.exists(path.cut)) dir.create(path.cut)
fnFs.cut <- file.path(path.cut, basename(fnFs))
fnRs.cut <- file.path(path.cut, basename(fnRs))

# Make flags for cutadapt
FWD.RC <- dada2:::rc(FWD)
REV.RC <- dada2:::rc(REV)
# Trim FWD and the reverse-complement of REV off of R1 (forward reads)
R1.flags <- paste("-g", FWD, "-a", REV.RC)
# Trim REV and the reverse-complement of FWD off of R2 (reverse reads)
R2.flags <- paste("-G", REV, "-A", FWD.RC)

# Run Cutadapt
# Cutadapt doesn't work from windows R to ubuntu.
# instead, we did this step in bash with a shell script:
    # dir_in = /mnt/d/16S/filtN
    # dir_out = /mnt/d/16S/cutadapt
    #
    # echo "Starting cutadapt"
    #
    # cd /mnt/d/16S/filtN/
    #
    #   for i in *R1.fastq
    # do
    # base=$(basename $i _R1.fastq)
    # tput setaf 6; echo "Using cutadapt on $base"
    # tput setaf 2
    # cutadapt \
    # -j 8 \
    # -g GTGCCAGCMGCCGCGGTAA -a ATTAGAWACCCBDGTAGTCC \
    # -G GGACTACHVGGGTWTCTAAT -A TTACCGCGGCKGCTGGCAC \
    # -n 2 \
    # -o /mnt/d/16S/cutadapt/${base}_R1.fastq \
    # -p /mnt/d/16S/cutadapt/${base}_R2.fastq \
    # ${base}_R1.fastq \
    # ${base}_R2.fastq
    # tput setaf 3; echo "Finished running cutadapt on $base"
    #
    # done
    #
    # echo "Done!"

rbind(FWD.ForwardReads = sapply(FWD.orients, primerHits, fn = fnFs.cut[[1]]),
    FWD.ReverseReads = sapply(FWD.orients, primerHits, fn = fnRs.cut[[1]]),
    REV.ForwardReads = sapply(REV.orients, primerHits, fn = fnFs.cut[[1]]),
    REV.ReverseReads = sapply(REV.orients, primerHits, fn = fnRs.cut[[1]]))

### Inspect read quality profiles ####

# Forward and reverse fastq filenames have format: SAMPLENAME_R1_001.fastq and SAMPLENAME_R2_001.fastq
fnFs <- sort(list.files(path.cut, pattern="_R1.fastq", full.names = TRUE))
fnRs <- sort(list.files(path.cut, pattern="_R2.fastq", full.names = TRUE))
# Extract sample names, assuming filenames have format: SAMPLENAME_XXX.fastq
sample.names <- sapply(strsplit(basename(fnFs), "_"), `[`, 1)

plotQualityProfile(fnFs[1:24])
plotQualityProfile(fnRs[1:24])


### Filter and trim ####

# Place filtered files in filtered/ subdirectory
filtFs <- file.path(path, "filtered", paste0(sample.names, "_F_filt.fastq.gz"))
```

```
filtRs <- file.path(path, "filtered", paste0(sample.names, "_R_filt.fastq.gz"))
names(filtFs) <- sample.names
names(filtRs) <- sample.names

# Filter and trim
# truncLen (c(fw_length, rv_length))
# maxN = 0, because Dada2 doesnt like N

  out <- filterAndTrim(fnFs, filtFs, fnRs, filtRs, truncLen=c(228,227),
                     maxN=0, minLen = 50, maxEE=c(2,2), truncQ=2, rm.phix=TRUE,
                     compress=TRUE, multithread=TRUE, verbose = TRUE) # On Windows set multithread=FALSE
  head(out)

# show quality again

plotQualityProfile(filtFs[1:24])
plotQualityProfile(filtRs[1:24])


### Learn the Error Rates ####

errF <- learnErrors(filtFs, multithread=TRUE, verbose = T)
errR <- learnErrors(filtRs, multithread=TRUE, verbose = T)

# Plot the error rates
plotErrors(errF, nominalQ=TRUE)
plotErrors(errR, nominalQ=TRUE)

### Run dada, Sample Inference ####

dadaFs <- dada(filtFs, err=errF, multithread=TRUE, verbose = T)
dadaRs <- dada(filtRs, err=errR, multithread=TRUE, verbose = T)

dadaFs[[1]]
dadaRs[[1]]

### Merging the F and R reads ####

mergers <- mergePairs(dadaFs, filtFs, dadaRs, filtRs, verbose=TRUE)
# Inspect the merger data.frame from the first sample
head(mergers[[1]])

### Constructing a sequence table ####

seqtab <- makeSequenceTable(mergers)
dim(seqtab)

# Inspect distribution of sequence lengths
table(nchar(getSequences(seqtab)))

# Some outliers detected (too long), so we cut off the product

seqtab2 <- seqtab[,nchar(colnames(seqtab)) %in% 250:256]
dim(seqtab2)

table(nchar(getSequences(seqtab2)))

# Removing Chimeras from the seqtab

seqtab.nochim <- removeBimeraDenovo(seqtab2, method="consensus", multithread=TRUE, verbose=TRUE)
dim(seqtab.nochim)

sum(seqtab.nochim)/sum(seqtab)

### Finally, we check how many reads made it through all these steps.
getN <- function(x) sum(getUniques(x))
track <- cbind(out, sapply(dadaFs, getN), sapply(dadaRs, getN), sapply(mergers, getN), rowSums(seqtab.nochim))
# If processing a single sample, remove the sapply calls: e.g. replace sapply(dadaFs, getN) with getN(dadaFs)
colnames(track) <- c("input", "filtered", "denoisedF", "denoisedR", "merged", "nonchim")
rownames(track) <- sample.names
head(track)


### Assign taxonomy ####
```

```
taxa_train_set <- assignTaxonomy(seqtab.nochim, "Taxonomy/16S/silva_nr99_v138_train_set.fa.gz", multithread=TRUE)
taxa <- addSpecies(taxa_train_set, "Taxonomy/16S/silva_species_assignment_v138.fa.gz")

### Save sequence table to csv
# Numbering of ASVs is just 1, 2, etc... without sorting.
sequences <- tax_table(taxa)
head(sequences)

#write.csv(sequences, file='Logs/16S_sequences.csv')

# preview the taxa
taxa.print <- taxa # Removing sequence rownames for display only
rownames(taxa.print) <- NULL
head(taxa.print)

### Phyloseq ####
library(phyloseq); packageVersion("phyloseq")
library(Biostrings); packageVersion("Biostrings")
library(ggplot2); packageVersion("ggplot2")
theme_set(theme_bw())

## Construct phyloseq dataframe

samples.out <- rownames(seqtab.nochim)
sample <- rep(c("1", "2", "3", "4"), times = 6)
treatment <- rep(c("B_brassicae",
            "Control",
            "D_radicum",
            "P_xylostella",
            "Rhizo",
            "Rhizo_Delia"), each = 4)
herbivory <- rep(c("AG_Herbivory",
            "Control",
            "BG_Herbivory",
            "AG_Herbivory",
            "Control",
            "BG_Herbivory"), each = 4)
Rhizo <- rep(c("Control",
            "Control",
            "Control",
            "Control",
            "Rhizo",
            "Rhizo"), each = 4)
Delia <- rep(c("Control",
            "Control",
            "D_radicum",
            "Control",
            "Control",
            "D_radicum"), each = 4)
samdf <- data.frame(Sample=sample,
             Treatment=treatment,
             Herbivory = herbivory,
             Rhizo = Rhizo,
             Delia = Delia)
rownames(samdf) <- samples.out

ps <- phyloseq(otu_table(seqtab.nochim, taxa_are_rows=FALSE),
        sample_data(samdf),
        tax_table(taxa))

# Change taxa names to short versions, but keep the old ones

dna <- Biostrings::DNAStringSet(taxa_names(ps))
names(dna) <- taxa_names(ps)
ps <- merge_phyloseq(ps, dna)
taxa_names(ps) <- paste0("ASV", seq(ntaxa(ps)))
ps

# load("Logs/16S_DaDa_phyloseq.Rdata")
# save.image(file = "Logs/16S_DaDa_phyloseq.Rdata") # save entire environment
save(ps, file = "Logs/phyloseq_16S.RData")  # save phyloseq object
```

## ITS_dada.R

```
# Shoot and root insect herbivory changes the plant rhizosphere microbiome and affects cabbage-insect interactions through plant-soil feedback
#
# This script is used for processing raw ITS reads using DADA2
# by Julia Friman and Peter Karssemeijer
#
#
#
# https://benjjneb.github.io/dada2/tutorial.html # DADA2 tutorial we followed
#
#

rm(list=ls())                                                            ## clear workspace ##

#::install("phyloseq")
#BiocManager::install("dada2")

library(dada2); packageVersion("dada2")
library(ShortRead); packageVersion("ShortRead")
library(Biostrings); packageVersion("Biostrings")

# Allow multithreading
library(RcppParallel)
setThreadOptions(numThreads = 8)
defaultNumThreads()

# Set working directory
setwd(dir = "my/favourite/folder")

### Set file names ####

path <- "Raw_Data/ITS"
list.files(path)

# Forward and reverse fastq filenames have format: SAMPLENAME_R1_001.fastq and SAMPLENAME_R2_001.fastq
fnFs <- sort(list.files(path, pattern="_R1.fastq", full.names = TRUE))
fnRs <- sort(list.files(path, pattern="_R2.fastq", full.names = TRUE))
# Extract sample names, assuming filenames have format: SAMPLENAME_XXX.fastq
sample.names <- sapply(strsplit(basename(fnFs), "_"), `[`, 1)

### Remove primers ###
FWD <- "GAACACAGCGAAATGTGA"  ## fITS9 primer
REV <- "TCCTCCGCTTATTGATATGC"  ## ITS4R primer

# produce all orientations #

allOrients <- function(primer) {
  # Create all orientations of the input sequence
  require(Biostrings)
  dna <- DNAString(primer)  # The Biostrings works w/ DNAString objects rather than character vectors
  orients <- c(Forward = dna, Complement = complement(dna), Reverse = reverse(dna),
               RevComp = reverseComplement(dna))
  return(sapply(orients, toString))  # Convert back to character vector
}
FWD.orients <- allOrients(FWD)

REV.orients <- allOrients(REV)

# Filter N

fnFs.filtN <- file.path(path, "filtN", basename(fnFs)) # Put N-filterd files in filtN/ subdirectory
fnRs.filtN <- file.path(path, "filtN", basename(fnRs))
filterAndTrim(fnFs, fnFs.filtN, fnRs, fnRs.filtN,
              maxN = 0,
              rm.phix=TRUE,
              compress=FALSE,
              multithread=TRUE,
              verbose = TRUE)

# Check the presence of primers
primerHits <- function(primer, fn) {
  # Counts number of reads in which the primer is found
  nhits <- vcountPattern(primer, sread(readFastq(fn)), fixed = FALSE)
```

```
  return(sum(nhits > 0))
}


rbind(FWD.ForwardReads = sapply(FWD.orients, primerHits, fn = fnFs.filtN[[1]]),
      FWD.ReverseReads = sapply(FWD.orients, primerHits, fn = fnRs.filtN[[1]]),
      REV.ForwardReads = sapply(REV.orients, primerHits, fn = fnFs.filtN[[1]]),
      REV.ReverseReads = sapply(REV.orients, primerHits, fn = fnRs.filtN[[1]]))


### Run Cutadapt! ####

path.cut <- file.path(path, "cutadapt")
if(!dir.exists(path.cut)) dir.create(path.cut)
fnFs.cut <- file.path(path.cut, basename(fnFs))
fnRs.cut <- file.path(path.cut, basename(fnRs))

# Make flags for cutadapt
FWD.RC <- dada2:::rc(FWD)
REV.RC <- dada2:::rc(REV)
# Trim FWD and the reverse-complement of REV off of R1 (forward reads)
R1.flags <- paste("-g", FWD, "-a", REV.RC)
# Trim REV and the reverse-complement of FWD off of R2 (reverse reads)
R2.flags <- paste("-G", REV, "-A", FWD.RC)

# Run Cutadapt
  # A 5' adapter is assumed to be ligated to the 5' end of your sequence of interest.
  # When such an adapter is found, the adapter sequence itself and the sequence preceding it
  # (if there is any) are trimmed.
    # In other words, if we only use the primer sequence, and another adapter is in front of it,
    # that will be removed as well!

# Cutadapt doesn't work from windows R to ubuntu.
# instead, we did this step in ubuntu with a shell script:
        # dir_in = /mnt/d/ITS/filtN
        # dir_out = /mnt/d/ITS/cutadapt
        #
        # echo "Starting cutadapt"
        #
        # cd /mnt/d/ITS/filtN/
        #
        #   for i in *R1.fastq
        # do
        # base=$(basename $i _R1.fastq)
        # tput setaf 6; echo "Using cutadapt on $base"
        # tput setaf 2
        # cutadapt \
        # -j 8 \
        # -g GAACACAGCGAAATGTGA -a GCATATCAATAAGCGGAGGA \
        # -G TCCTCCGCTTATTGATATGC -A TCACATTTCGCTGTGTTC \
        # -n 2 \
        # -o /mnt/d/16S/cutadapt/${base}_R1.fastq \
        # -p /mnt/d/16S/cutadapt/${base}_R2.fastq \
        # ${base}_R1.fastq \
        # ${base}_R2.fastq
        # tput setaf 3; echo "Finished running cutadapt on $base"
        #
        # done
        #
        # echo "Done!"


rbind(FWD.ForwardReads = sapply(FWD.orients, primerHits, fn = fnFs.cut[[1]]),
      FWD.ReverseReads = sapply(FWD.orients, primerHits, fn = fnRs.cut[[1]]),
      REV.ForwardReads = sapply(REV.orients, primerHits, fn = fnFs.cut[[1]]),
      REV.ReverseReads = sapply(REV.orients, primerHits, fn = fnRs.cut[[1]]))

### Inspect read quality profiles ####

# Forward and reverse fastq filenames have format: SAMPLENAME_R1_001.fastq and SAMPLENAME_R2_001.fastq
fnFs <- sort(list.files(path.cut, pattern="_R1.fastq", full.names = TRUE))
fnRs <- sort(list.files(path.cut, pattern="_R2.fastq", full.names = TRUE))
# Extract sample names, assuming filenames have format: SAMPLENAME_XXX.fastq
sample.names <- sapply(strsplit(basename(fnFs), "_"), `[`, 1)
```

```
plotQualityProfile(fnFs[1:24])
plotQualityProfile(fnRs[1:24])


### Filter and trim ####

# Place filtered files in filtered/ subdirectory
filtFs <- file.path(path, "filtered", paste0(sample.names, "_F_filt.fastq.gz"))
filtRs <- file.path(path, "filtered", paste0(sample.names, "_R_filt.fastq.gz"))
names(filtFs) <- sample.names
names(filtRs) <- sample.names

# Filter and trim
# We don't use Trunlen here, because for ITS we want maximum overlap
# maxN = 0, because Dada2 doesnt like N

  out <- filterAndTrim(fnFs, filtFs, fnRs, filtRs,
                     maxN=0, minLen = 50, maxEE=c(2,2), truncQ=2, rm.phix=TRUE,
                     compress=TRUE, multithread=TRUE, verbose = TRUE) # On Windows set multithread=FALSE

  head(out)

  # show quality again

plotQualityProfile(filtFs[1:24])
plotQualityProfile(filtRs[1:24])


### Learn the Error Rates ####

errF <- learnErrors(filtFs, multithread=TRUE, verbose = T)
errR <- learnErrors(filtRs, multithread=TRUE, verbose = T)

# Plot the error rates
plotErrors(errF, nominalQ=TRUE)
plotErrors(errR, nominalQ=TRUE)

### Run dada, Sample Inference ####

dadaFs <- dada(filtFs, err=errF, multithread=TRUE, verbose = T)
dadaRs <- dada(filtRs, err=errR, multithread=TRUE, verbose = T)

dadaFs[[1]]
dadaRs[[1]]

### Merging the F and R reads ####

mergers <- mergePairs(dadaFs, filtFs, dadaRs, filtRs, verbose=TRUE)
# Inspect the merger data.frame from the first sample
head(mergers[[1]])

### Constructing a sequence table ####

seqtab <- makeSequenceTable(mergers)
dim(seqtab)

# Inspect distribution of sequence lengths
table(nchar(getSequences(seqtab)))

# Removing Chimeras from the seqtab

seqtab.nochim <- removeBimeraDenovo(seqtab, method="consensus", multithread=TRUE, verbose=TRUE)
dim(seqtab.nochim)

sum(seqtab.nochim)/sum(seqtab)

### Finally, we check how many reads made it through all these steps.
getN <- function(x) sum(getUniques(x))
track <- cbind(out, sapply(dadaFs, getN), sapply(dadaRs, getN), sapply(mergers, getN), rowSums(seqtab.nochim))
# If processing a single sample, remove the sapply calls: e.g. replace sapply(dadaFs, getN) with getN(dadaFs)
colnames(track) <- c("input", "filtered", "denoisedF", "denoisedR", "merged", "nonchim")
rownames(track) <- sample.names
head(track)
```

```
### Assign taxonomy ####

unite.ref <- "Taxonomy/ITS/sh_general_release_s_04.02.2020/sh_general_release_dynamic_s_04.02.2020.fasta"  # CHANGE ME to location on your machine
taxa <- assignTaxonomy(seqtab.nochim, unite.ref, multithread = TRUE, tryRC = TRUE)

# preview the taxa
taxa.print <- taxa # Removing sequence rownames for display only
rownames(taxa.print) <- NULL
head(taxa.print)

### Phyloseq ####
library(phyloseq); packageVersion("phyloseq")
library(Biostrings); packageVersion("Biostrings")
library(ggplot2); packageVersion("ggplot2")
theme_set(theme_bw())

## Construct phyloseq dataframe

samples.out <- rownames(seqtab.nochim)
sample <- rep(c("1", "2", "3", "4"), times = 6)
treatment <- rep(c("B_brassicae",
                "Control",
                "D_radicum",
                "P_xylostella",
                "Rhizo",
                "Rhizo_Delia"), each = 4)
herbivory <- rep(c("AG_Herbivory",
                "Control",
                "BG_Herbivory",
                "AG_Herbivory",
                "Control",
                "BG_Herbivory"), each = 4)
Rhizo <- rep(c("Control",
             "Control",
             "Control",
             "Control",
             "Rhizo",
             "Rhizo"), each = 4)
Delia <- rep(c("Control",
             "Control",
             "D_radicum",
             "Control",
             "Control",
             "D_radicum"), each = 4)
samdf <- data.frame(Sample=sample,
                Treatment=treatment,
                Herbivory = herbivory,
                Rhizo = Rhizo,
                Delia = Delia)


rownames(samdf) <- samples.out

ps <- phyloseq(otu_table(seqtab.nochim, taxa_are_rows=FALSE),
             sample_data(samdf),
             tax_table(taxa))

# Change taxa names to short versions, but keep the old ones

dna <- Biostrings::DNAStringSet(taxa_names(ps))
names(dna) <- taxa_names(ps)
ps <- merge_phyloseq(ps, dna)
taxa_names(ps) <- paste0("ASV", seq(ntaxa(ps)))
ps

tax_table(ps)

# save.image(file = "Logs/ITS_DaDa_phyloseq.Rdata") # save entire environment
save(ps, file = "Logs/phyloseq_ITS.RData")
```

## 16S_analysis.R

```
# Shoot and root insect herbivory changes the plant rhizosphere microbiome and affects cabbage-insect interactions through plant-soil feedback
#
# This script is used for analysis of 16S data
# includes ordination, relative abundance, and differential ASV analysis
#
# by Julia Friman and Peter Karssemeijer
#

rm(list=ls())                                                                                                  ## clear workspace ##

# Set working directory
setwd(dir = "my/favourite/folder")

# Load phyloseq object

load("Logs/phyloseq_16S.RData")

# Allow multithreading
library(RcppParallel)
setThreadOptions(numThreads = 8)
defaultNumThreads()

# Load required packages

library(RVAideMemoire)
library(metagenomeSeq)
library(phyloseq)
library(tidyr)
library(viridis)
library(RColorBrewer)
library(ggplot2)
library(DESeq2); packageVersion("DESeq2")

##### Cut off ASVs with below average Effective Samples ####
# Method in accordance with metagenomeseq vignette

# Transform phyloseq object into an MRexperiment object

obj = phyloseq_to_metagenomeSeq(ps)

#Calculate effective samples
objp=cumNormStat(obj,pFlag = TRUE,main="Rhizosphere data")
obj2=cumNorm(obj,p=objp)
treatment=pData(obj2)$Treatment
settings=zigControl(tol=1e-10,maxit = 10,verbose = TRUE)
mod=model.matrix(~treatment)
colnames(mod)=levels(treatment)
res=fitZig(obj = obj2,mod = mod,control = settings)
esesam<-calculateEffectiveSamples(res)
esesam<-as.data.frame(esesam)

# take out below-average ASV's
average_effective_size <- mean(esesam[,])
cutoff_asv <- esesam[]<=average_effective_size
asv_to_remove <- esesam[cutoff_asv, , drop=FALSE]

pseff <- prune_taxa(taxa_names(ps)[!(taxa_names(ps) %in% rownames(asv_to_remove))], ps)
ps <- pseff

# Remove non-informative groups
# Archae, Mitochondria, Chloroplasts
# write.csv(tax_table(pseff),"Logs/16S_Taxonomyeff.csv")
sel <- read.csv("Logs/16S_Taxonomyeff.csv") # In excel, we manually took out these groups
rownames(sel) <- sel$ASV

pseff <- prune_taxa(taxa_names(pseff)[(taxa_names(pseff) %in% rownames(sel))], pseff)

#########################################################################################

objeffclean = phyloseq_to_metagenomeSeq(pseff)

#Calculate normalization factor and export normalized count matrix
peff=cumNormStatFast(objeffclean)
```

```
obj3=cumNorm(objeffclean,p=peff)
mateff=MRcounts(obj3,norm = TRUE,log=TRUE)

#Construct new phyloseq object with normalized OTU table
pseffnorm <- phyloseq(otu_table(t(mateff), taxa_are_rows=FALSE), sample_data(pseff), tax_table(pseff))

##########################################################################################
# Ordination

# Beta dispersion estimates (assumptions ordination)
# within group variation. For PERMANOVA we assume homogeneity of variance within groups.
library(vegan)

ps_distance <- phyloseq::distance(pseffnorm, "bray")
ps_beta <- betadisper(ps_distance, group = sample_data(pseffnorm)$Treatment)

anova(ps_beta)
permutest(ps_beta, permutations = 99999)
plot(ps_beta)
   # Assumptions are met (non significant difference in betadispersion)

ord.PCoA.bray<-ordinate(pseffnorm,method="RDA",distance="bray")


plot_ordination(title = "16S grouped by Treatment",pseffnorm,ord.PCoA.bray,color = "Treatment")+scale_colour_viridis_d(direction = -1)+ geom_point(size=4)

plot <- plot_ordination(pseffnorm,ord.PCoA.bray,color = "Herbivory", shape = "Treatment") +
            geom_point(size=4, stroke = 1.5)+
            scale_colour_manual(values=c('darkcyan','firebrick', 'gray30'))+
            scale_shape_manual(values=c(0, 1, 2, 15,16,17))+
            theme_bw(base_size = 15)

plot$layers <- plot$layers[-1]
plot
  #ggsave("Figures/16S_PCA.pdf")

# PERMANOVA
adonis(phyloseq::distance(pseffnorm, "bray")
      ~ Treatment,
      permutations = 99999,
      as(sample_data(pseffnorm),
        "data.frame"))

adonis(phyloseq::distance(pseffnorm, "bray")
      ~ Herbivory,
      permutations = 99999,
      as(sample_data(pseffnorm),
        "data.frame"))

# Posthoc (Hu et al 2018, supp3, methods by Klaus Schlaeppi)
posthoc_treatment <- pairwise.perm.manova(phyloseq::distance(pseffnorm, "bray"),
                fact = sample_data(pseffnorm)$Treatment,
                progress = T,
                nperm = 99999,
                p.method = "fdr",
                F = T,
                R2 = T)
posthoc_treatment$p.value
posthoc_treatment$F.value
posthoc_treatment$R2.value


posthoc_herbivory <- pairwise.perm.manova(phyloseq::distance(pseffnorm, "bray"),
                fact = sample_data(pseffnorm)$Herbivory,
                progress = T,
                nperm = 99999,
                p.method = "fdr",
                F = T,
                R2 = T)
posthoc_herbivory$p.value
posthoc_herbivory$F.value
posthoc_herbivory$R2.value

##########################################################################################
# Stacked graphs relative abundance
```

```
pseffnorm.phylum <- tax_glom(pseffnorm, taxrank="Phylum", NArm=FALSE)
pseffnorm.phylum <- merge_samples(pseffnorm.phylum, group = "Treatment", fun=mean)
tax_table(pseffnorm.phylum)
otu_table(pseffnorm.phylum)


alltax <- names(sort(taxa_sums(pseffnorm.phylum), decreasing=TRUE))[1:22]
ps.alltax <- transform_sample_counts(pseffnorm.phylum, function(OTU) OTU/sum(OTU))
ps.alltax <- prune_taxa(alltax, ps.alltax)
theme_set(theme_classic())

library(RColorBrewer)
color = kelly(n=21)


p = plot_bar(title = "16S Relative abundance",ps.alltax,  fill="Phylum") + scale_y_continuous(expand = c(0,0)) +
  scale_x_discrete(expand = c(0,0)) +
  scale_fill_manual(values = color)

uniqueVectorOfLevelsInOrder <- c("Control", "Rhizo", "B_brassicae", "P_xylostella", "D_radicum", "Rhizo_Delia")
p$data$Sample <- factor(p$data$Sample, levels = uniqueVectorOfLevelsInOrder)
p

#ggsave("Figures/16S_Phylum_rel_abundance.pdf")


##########################################################################################
# Differential ASV analysis

# phyloseq to deseq2

dds <- phyloseq_to_deseq2(pseff, ~Treatment)
dds$Treatment <- relevel(dds$Treatment, "Control")

# run DESeq2

dds <- DESeq(dds, test="Wald")

# Set padj for pairwise comparisons
padj <- 0.05

# extract results

# Brevicoryne vs Control
res_Bb <- results(dds, alpha = padj, contrast = c("Treatment", "B_brassicae", "Control"))
summary(res_Bb)
sel_Bb <- res_Bb$padj <= padj &
  ! is.na(res_Bb$padj)
summary(res_Bb[sel_Bb,])
res_Bb.filtered <- res_Bb[sel_Bb,]

# Delia vs Control
res_Dr <- results(dds, alpha = padj, contrast = c("Treatment", "D_radicum", "Control"))
summary(res_Dr)
sel_Dr <- res_Dr$padj <= padj &
  ! is.na(res_Dr$padj)
summary(res_Dr[sel_Dr,])
res_Dr.filtered <- res_Dr[sel_Dr,]

# Plutella vs Control
res_Px <- results(dds, alpha = padj, contrast = c("Treatment", "P_xylostella", "Control"))
summary(res_Px)
sel_Px <- res_Px$padj <= padj &
  ! is.na(res_Px$padj)
summary(res_Px[sel_Px,])
res_Px.filtered <- res_Px[sel_Px,]

# Rhizo vs Control
res_Rh <- results(dds, alpha = padj, contrast = c("Treatment", "Rhizo", "Control"))
summary(res_Rh)
sel_Rh <- res_Rh$padj <= padj &
  ! is.na(res_Rh$padj)
summary(res_Rh[sel_Rh,])
res_Rh.filtered <- res_Rh[sel_Rh,]
```

```
# Rhizo_Delia vs Control
res_RD <- results(dds, alpha = padj, contrast = c("Treatment", "Rhizo_Delia", "Control"))
summary(res_RD)
sel_RD <- res_RD$padj <= padj &
  ! is.na(res_RD$padj)
summary(res_RD[sel_RD,])
res_RD.filtered <- res_RD[sel_RD,]

# Differential expressed ASV list
# Rownames of each treatment
Bb <-rownames(res_Bb.filtered)
Px <-rownames(res_Px.filtered)
Dr <-rownames(res_Dr.filtered)
Rh <-rownames(res_Rh.filtered)
RD <-rownames(res_RD.filtered)

# Combine these lists and take out duplicated
Common_ASV <- unique(c(Bb,Px,Dr,Rh,RD))

#  Calculate normalized counts
nt <- normTransform(dds) # defaults to log2(x+1)
Heatmap_data <- assay(nt)[Common_ASV, ]

colData <- as.data.frame(c(as.data.frame(colData(dds)$Treatment), as.data.frame(colData(dds)$Herbivory))) # Make sample data file for heatmap

colnames(colData) <- c("Treatment", "Herbivory")
rownames(colData) <- colnames(Heatmap_data)

# Change names to Class - Genus (manually in excel)
Tax_table <- tax_table(pseff)
Tax_table_heatmap <- Tax_table[rownames(Heatmap_data),]
#write.csv(Tax_table_heatmap, "Logs/16S_tax_table_heatmap.csv")
Rownames_heatmap <- read.csv(file="Logs/16S_tax_table_heatmap.csv", header = T)
Rownames_heatmap <- Rownames_heatmap$Rownames

# Parameters to standardize heatmaps
library(RColorBrewer)
library(pheatmap)
library(pals)

breaksList = seq(0, 15, by = 0.1) # Determines axis and color scale
cell_width = 10     # Width of cells
cell_height = 8       # Height of cells
display_numbers = F  # Whether to show numbers in cells
color = rev(ocean.haline(150))

ASV_Heatmap <- pheatmap(Heatmap_data,
                        #filename = "Figures/16S_heatmap_green_ASV.pdf",
                        annotation_col = colData,
                        main = "16S - log2(counts + 1)",
                        #labels_row = Rownames_heatmap,
                        fontsize_row = 8,
                        border_color = "grey60",
                        angle_col = 45,
                        cluster_rows = T,
                        cluster_cols = T,
                        na_col = "white",
                        cellwidth = cell_width,
                        cellheight = cell_height,
                        display_numbers = display_numbers,
                        color = color,
                        breaks = breaksList) # Sets the breaks of the color scale as in breaksList
```

# ITS_analysis.R

```
# Shoot and root insect herbivory changes the plant rhizosphere microbiome and affects cabbage-insect interactions through plant-soil feedback
#
# This script is used for analysis of ITS data
# includes ordination, relative abundance, and differential ASV analysis
#
# by Julia Friman and Peter Karssemeijer
#

rm(list=ls())                                                                    ## clear workspace ##

# Set working directory
setwd(dir = "my/favourite/folder")

# Load phyloseq object

load("Logs/phyloseq_16S.RData")

# Allow multithreading
library(RcppParallel)
setThreadOptions(numThreads = 8)
defaultNumThreads()

# Load required packages

library(RVAideMemoire)
library(metagenomeSeq)
library(phyloseq)
library(tidyr)
library(RColorBrewer)
library(ggplot2)
library(DESeq2); packageVersion("DESeq2")

##### Cut off ASVs with below average Effective Samples ####
# Method in accordance with metagenomeseq vignette

# Transform phyloseq object into an MRexperiment object

obj = phyloseq_to_metagenomeSeq(ps)

#Calculate effective sample sizes
objp=cumNormStat(obj,pFlag = TRUE,main="Rhizosphere data")
obj2=cumNorm(obj,p=objp)
treatment=pData(obj2)$Treatment
settings=zigControl(tol=1e-10,maxit = 10,verbose = TRUE)
mod=model.matrix(~treatment)
colnames(mod)=levels(treatment)
res=fitZig(obj = obj2,mod = mod,control = settings)
esesam<-calculateEffectiveSamples(res)
esesam<-as.data.frame(esesam)

# take out below-average ASV's
average_effective_size <- mean(esesam[,])
cutoff_asv <- esesam[]<=average_effective_size
asv_to_remove <- esesam[cutoff_asv, , drop=FALSE]

pseff <- prune_taxa(taxa_names(ps)[!(taxa_names(ps) %in% rownames(asv_to_remove))], ps)
ps <- pseff

# For fungi, groups specified to only kingdom were omitted (about 30 ASVs)
#write.csv(tax_table(pseff),"Logs/Taxonomyeff_ITS.csv")
sel <- read.csv("Logs/Taxonomyeff_ITS.csv") # In excel, we manually took out these groups
rownames(sel) <- sel$X

pseff <- prune_taxa(taxa_names(pseff)[(taxa_names(pseff) %in% rownames(sel))], pseff)

##########################################################################################

objeffclean = phyloseq_to_metagenomeSeq(pseff)

#Calculate normalization factor and export normalized count matrix
peff=cumNormStatFast(objeffclean)
obj3=cumNorm(objeffclean,p=peff)
mateff=MRcounts(obj3,norm = TRUE,log=TRUE)
```

```
#Construct new phyloseq object with normalized OTU table
pseffnorm <- phyloseq(otu_table(t(mateff), taxa_are_rows=FALSE), sample_data(pseff), tax_table(pseff))

##########################################################################################
# Ordination

# Beta dispersion estimates (assumptions ordination)
# within group variation. For PERMANOVA we assume homogeneity of variance within groups.
library(vegan)
ps_distance <- phyloseq::distance(pseffnorm, "bray")
ps_beta <- betadisper(ps_distance, group = sample_data(pseffnorm)$Treatment)

anova(ps_beta)
permutest(ps_beta, permutations = 99999)
plot(ps_beta)
   # Assumptions are met (non significant difference in betadispersion)

ord.PCoA.bray<-ordinate(pseffnorm,method="RDA",distance="bray")

plot <- plot_ordination(pseffnorm,ord.PCoA.bray,color = "Herbivory", shape = "Treatment", axes=c(1,2)) +
  geom_point(size=4, stroke = 1.5)+
  scale_colour_manual(values=c('darkcyan','firebrick', 'gray30'))+
  scale_shape_manual(values=c(0, 1, 2, 15,16,17))+
  theme_bw(base_size = 15)

plot$layers <- plot$layers[-1]
plot
#ggsave("Figures/ITS_PCA.pdf")

plot_ordination(title = "PCA ITS colored by Herbivory",pseffnorm,ord.PCoA.bray,color = "Herbivory")+scale_colour_viridis_d(direction = -1)+ geom_point(size=4)
plot_ordination(pseffnorm,ord.PCoA.bray,color = "Treatment")+scale_colour_viridis_d(direction = -1)+stat_ellipse(size=1)+ geom_point(size=1)

# PERMANOVA
adonis(phyloseq::distance(pseffnorm, "bray")
      ~ Treatment,
      permutations = 99999,
      as(sample_data(pseffnorm),
        "data.frame"))

adonis(phyloseq::distance(pseffnorm, "bray")
      ~ Herbivory,
      permutations = 99999,
      as(sample_data(pseffnorm),
        "data.frame"))

# Posthoc (Hu et al 2018, supp3, methods by Klaus Schlaeppi)
posthoc_treatment <- pairwise.perm.manova(phyloseq::distance(pseffnorm, "bray"),
                                 fact = sample_data(pseffnorm)$Treatment,
                                 progress = F,
                                 nperm = 99999,
                                 p.method = "fdr",
                                 F = T,
                                 R2 = T)
posthoc_treatment$p.value
posthoc_treatment$F.value
posthoc_treatment$R2.value


posthoc_herbivory <- pairwise.perm.manova(phyloseq::distance(pseffnorm, "bray"),
                                 fact = sample_data(pseffnorm)$Herbivory,
                                 progress = F,
                                 nperm = 99999,
                                 p.method = "fdr",
                                 F = T,
                                 R2 = T)
posthoc_herbivory$p.value
posthoc_herbivory$F.value
posthoc_herbivory$R2.value


##########################################################################################
# Stacked graphs relative abundance

#report relative abundance of genus NA
```

```
pseffnorm.phylum <- tax_glom(pseffnorm, taxrank="Phylum", NArm=FALSE)
pseffnorm.phylum <- merge_samples(pseffnorm.phylum, group = "Treatment", fun=mean)
tax_table(pseffnorm.phylum)
otu_table(pseffnorm.phylum)


alltax <- names(sort(taxa_sums(pseffnorm.phylum), decreasing=TRUE))[1:5]
ps.alltax <- transform_sample_counts(pseffnorm.phylum, function(OTU) OTU/sum(OTU))
ps.alltax <- prune_taxa(alltax, ps.alltax)

theme_set(theme_classic())
library(RColorBrewer)
color = rev(kelly(n=21))

p = plot_bar(title = "ITS relative abundance", ps.alltax,  fill="Phylum") + scale_y_continuous(expand = c(0,0)) +
  scale_x_discrete(expand = c(0,0)) +
  scale_fill_manual(values = color)

uniqueVectorOfLevelsInOrder <- c("Control", "Rhizo", "B_brassicae", "P_xylostella", "D_radicum", "Rhizo_Delia")
p$data$Sample <- factor(p$data$Sample, levels = uniqueVectorOfLevelsInOrder)
p

#ggsave("Figures/ITS_Phylum_rel_abundance.pdf")


#########################################################################################
# Differential ASV analysis

# phyloseq to deseq2
# phyloseq to deseq2

dds <- phyloseq_to_deseq2(pseff, ~Treatment)
dds$Treatment <- relevel(dds$Treatment, "Control")

# run DESeq2

dds <- DESeq(dds, test="Wald")

# Set padj for pairwise comparisons
padj <- 0.05

# extract results

# Brevicoryne vs Control
res_Bb <- results(dds, alpha = padj, contrast = c("Treatment", "B_brassicae", "Control"))
summary(res_Bb)
sel_Bb <- res_Bb$padj <= padj &
  ! is.na(res_Bb$padj)
summary(res_Bb[sel_Bb,])
res_Bb.filtered <- res_Bb[sel_Bb,]

# Delia vs Control
res_Dr <- results(dds, alpha = padj, contrast = c("Treatment", "D_radicum", "Control"))
summary(res_Dr)
sel_Dr <- res_Dr$padj <= padj &
  ! is.na(res_Dr$padj)
summary(res_Dr[sel_Dr,])
res_Dr.filtered <- res_Dr[sel_Dr,]

# Plutella vs Control
res_Px <- results(dds, alpha = padj, contrast = c("Treatment", "P_xylostella", "Control"))
summary(res_Px)
sel_Px <- res_Px$padj <= padj &
  ! is.na(res_Px$padj)
summary(res_Px[sel_Px,])
res_Px.filtered <- res_Px[sel_Px,]

# Rhizo vs Control
res_Rh <- results(dds, alpha = padj, contrast = c("Treatment", "Rhizo", "Control"))
summary(res_Rh)
sel_Rh <- res_Rh$padj <= padj &
  ! is.na(res_Rh$padj)
summary(res_Rh[sel_Rh,])
res_Rh.filtered <- res_Rh[sel_Rh,]
```

```
# Rhizo_Delia vs Control
res_RD <- results(dds, alpha = padj, contrast = c("Treatment", "Rhizo_Delia", "Control"))
summary(res_RD)
sel_RD <- res_RD$padj <= padj &
  ! is.na(res_RD$padj)
summary(res_RD[sel_RD,])
res_RD.filtered <- res_RD[sel_RD,]

# Differential expressed ASV list
# Rownames of each treatment
Bb <-rownames(res_Bb.filtered)
Px <-rownames(res_Px.filtered)
Dr <-rownames(res_Dr.filtered)
Rh <-rownames(res_Rh.filtered)
RD <-rownames(res_RD.filtered)

# Combine these lists and take out duplicated
Common_ASV <- unique(c(Bb,Px,Dr,Rh,RD))

#  Calculate normalized counts
nt <- normTransform(dds) # defaults to log2(x+1)
Heatmap_data <- assay(nt)[Common_ASV, ]

colData <- as.data.frame(c(as.data.frame(colData(dds)$Treatment), as.data.frame(colData(dds)$Herbivory))) # Make sample data file for heatmap

colnames(colData) <- c("Treatment", "Herbivory")
rownames(colData) <- colnames(Heatmap_data)

# Change names to Class - Genus (manually in excel)
Tax_table <- tax_table(pseff)
Tax_table_heatmap <- Tax_table[rownames(Heatmap_data),]
#write.csv(Tax_table_heatmap, "Logs/ITS_tax_table_heatmap.csv")
Rownames_heatmap <- read.csv(file="Logs/ITS_tax_table_heatmap.csv", header = T)
Rownames_heatmap <- Rownames_heatmap$Rownames

# Parameters to standardize heatmaps
library(RColorBrewer)
library(dichromat)
library(pheatmap)
library(pals)

breaksList = seq(0, 15, by = 0.1) # Determines axis and color scale
cell_width = 10     # Width of cells
cell_height = 8       # Height of cells
display_numbers = F  # Whether to show numbers in cells
color = rev(ocean.haline(150))

ASV_Heatmap <- pheatmap(Heatmap_data,
                    #filename = "Figures/ITS_heatmap_green_ASV.pdf",
                    annotation_col = colData,
                    main = "Fungal ITS, log2(counts + 1)",
                    labels_row = Rownames_heatmap,
                    fontsize_row = 8,
                    border_color = "grey60",
                    angle_col = 45,
                    cluster_rows = T,
                    cluster_cols = T,
                    na_col = "white",
                    cellwidth = cell_width,
                    cellheight = cell_height,
                    display_numbers = display_numbers,
                    color = color,
                    breaks = breaksList) # Sets the breaks of the color scale as in breaksList
```
